# Conversion of nanoscale topographical information of cluster-assembled zirconia surfaces into mechanotransductive events promotes neuronal differentiation

**DOI:** 10.1186/s12951-016-0171-3

**Published:** 2016-03-09

**Authors:** Carsten Schulte, Simona Rodighiero, Martino Alfredo Cappelluti, Luca Puricelli, Elisa Maffioli, Francesca Borghi, Armando Negri, Elisa Sogne, Massimiliano Galluzzi, Claudio Piazzoni, Margherita Tamplenizza, Alessandro Podestà, Gabriella Tedeschi, Cristina Lenardi, Paolo Milani

**Affiliations:** CIMAINA, Dipartimento di Fisica, Università degli Studi di Milano, via Celoria 16, Milan, 20133 Italy; SEMM European School of Molecular Medicine, Via Adamello 16, Milan, 20139 Italy; DIVET, Università degli Studi di Milano, via Celoria 10, Milan, 20133 Italy; Fondazione Filarete, via le Ortles 22/4, Milan, 20139 Italy

**Keywords:** Mechanotransduction, Focal adhesion, Biomaterial, Integrin, Atomic force microscopy, Biophysics, Cell adhesion, Proteomics

## Abstract

**Background:**

Thanks to mechanotransductive components cells are competent to perceive nanoscale topographical features of their environment and to convert the immanent information into corresponding physiological responses. Due to its complex configuration, unraveling the role of the extracellular matrix is particularly challenging. Cell substrates with simplified topographical cues, fabricated by top-down micro- and nanofabrication approaches, have been useful in order to identify basic principles. However, the underlying molecular mechanisms of this conversion remain only partially understood.

**Results:**

Here we present the results of a broad, systematic and quantitative approach aimed at understanding how the surface nanoscale information is converted into cell response providing a profound causal link between mechanotransductive events, proceeding from the cell/nanostructure interface to the nucleus. We produced nanostructured ZrO_2_ substrates with disordered yet controlled topographic features by the bottom-up technique supersonic cluster beam deposition, i.e. the assembling of zirconia nanoparticles from the gas phase on a flat substrate through a supersonic expansion. We used PC12 cells, a well-established model in the context of neuronal differentiation. We found that the cell/nanotopography interaction enforces a nanoscopic architecture of the adhesion regions that affects the focal adhesion dynamics and the cytoskeletal organization, which thereby modulates the general biomechanical properties by decreasing the rigidity of the cell. The mechanotransduction impacts furthermore on transcription factors relevant for neuronal differentiation (e.g. CREB), and eventually the protein expression profile. Detailed proteomic data validated the observed differentiation. In particular, the abundance of proteins that are involved in adhesome and/or cytoskeletal organization is striking, and their up- or downregulation is in line with their demonstrated functions in neuronal differentiation processes.

**Conclusion:**

Our work provides a deep insight into the molecular mechanotransductive mechanisms that realize the conversion of the nanoscale topographical information of SCBD-fabricated surfaces into cellular responses, in this case neuronal differentiation. The results lay a profound cell biological foundation indicating the strong potential of these surfaces in promoting neuronal differentiation events which could be exploited for the development of prospective research and/or biomedical applications. These applications could be e.g. tools to study mechanotransductive processes, improved neural interfaces and circuits, or cell culture devices supporting neurogenic processes.

**Electronic supplementary material:**

The online version of this article (doi:10.1186/s12951-016-0171-3) contains supplementary material, which is available to authorized users.

## Background

Cells are capable of sensing, in a surprisingly precise manner, nanoscale topographical features and mechanical characteristics of the microenvironment they interact with, mainly via integrin-mediated adhesion sites which serve as mechanoreceptors [1–4].

The conversion of these physical signals (structural and mechanical cues) into a modulation of the cellular (biochemical) responses is defined as mechanotransduction [5, 6]. The general meaning of this concept is that the stiffness and the topography of the extracellular matrix (ECM) [2, 4, 7] influence the architecture and composition of adhesions sites (e.g. integrin clustering) which feedbacks on the force transmission, cytoskeletal organization and mechanical properties (e.g. actomyosin network configuration) of the cell. The variation of the cellular biophysical state impacts on the nuclear architecture and mechanosensitive transcription factors which eventually modulate the cellular program or even the cell fate [1–6]. Mechanotransduction involves different molecules and/or cellular components, i.e. the ECM, channels, focal complexes/adhesions (FC or FA), the actomyosin network, transcription factors and the nucleus [1–9]. Cellular mechanosensing and mechanotransduction have been shown to play an important role in differentiation processes [2, 6, 9, 10], in particular also in a neuronal context. Especially the growth cones of neuronal cells constantly explore the status of their microenvironmental surroundings which in turn has a strong impact on the behavior (growth cone advancement or retraction/guidance) and differentiation/maturation events of these cells [11].

Many aspects, in particular the role of the ECM nanoscale topography and architecture in determining the mechanotransductive signaling, are still poorly understood due to the extremely high structural and functional complexity [2, 4, 7].

In order to dissect the fundamental factors concurring to the building of ECM nanotopographic complexity and to identify the structural ingredients for the mimicking of the in vivo ECM characteristics, a huge effort has been concentrated on the fabrication of artificial substrates where micron- and nanoscale features can be precisely controlled and mixed [2, 4, 9, 12].

In the case of neuronal cells, it has been demonstrated that they have a nanoscale sensitivity for environmental surface features and that their cellular activities are strongly affected by the interaction with these features [11–15]. Several studies suggest that the nanoscale topography obtained e.g. by the use of electrospun fibers [16], or ordered nanopatterns [17–20] can contribute to a modulation of neuronal differentiation processes.

Gaining a reliable and reproducible control of the topographic surface features on the nanoscale level to mimic the complex structure of the ECM is a daunting challenge: in the last decades a reductionist approach has been adopted consisting in the micro- and nanofabrication of simple basic motifs such as grooves, pillars, dots with different dimensions and pitches in order to reproduce and to recapitulate the elemental topographical cues that may influence the cell behavior [2, 4, 9, 12]. This approach has also been dictated by the fact that the vast majority of micro- and nanofabrication techniques were taken from the microelectronic or from the molecular electronic world (top-down lithographic approaches, micromolding, nanoimprint, etc.). This restricted also the choice of the substrate materials to those typically used in these contexts. In general these high-precision fabrication methods are quite expensive and difficult to scale-up [4, 12]. Most importantly it is yet to be demonstrated that starting from simple topographical motifs one can reconstruct realistically the ECM complexity [2].

As an alternative, methods exploiting chemical or physical etching are largely used in the production of implants since they provide the possibility of obtaining disordered surfaces at the nano- and microscale over large surfaces of different metallic materials [4, 12], however the tuning to the best performing surface topography in terms of cell adhesion and differentiation is based essentially on a trial-and-error approach with no predictive quantitative evaluation of the topographical features inducing the observed cell behavior [2, 4, 12].

There is still an eminent need for an in-depth insight of the proposed molecular mechanisms of mechanotransduction, even more in the context of biomaterials highlighted in this work, to realize a reliable, efficient and intelligent development of potential research and/or medical applications [2, 4, 9, 12]. Here we present the results of a broad, systematic and quantitative interdisciplinary approach aimed at capturing the complexity of the mechanotransductive signaling cascade provoked by cellular interaction with nanostructured zirconia substrates with tailored and reproducible nanoscale topography. Zirconia is a biocompatible material used in various clinical applications (i.e. for dental and orthopaedic prostheses), especially due to its favorable chemical and structural properties [21].

In this work the approach for the production of nanostructured ZrO_2_ substrates with disordered yet controlled topographic features is based on the assembling of zirconia nanoparticles, produced in the gas phase and accelerated in a supersonic expansion, on a flat substrate (Supersonic Cluster Beam Deposition, SCBD) [22]. This bottom-up assembling technique produces nanostructured films obtained by randomly distributed clusters, thus creating a nanoscale topography whose roughness can be accurately controlled and varied in a reproducible manner [23]. This very precise and reproducible control over nanoscale topography can be easily obtained over macroscopic areas which is a necessary requirement for the large number of experiments performed in this study.

As cell model to study the neuronal differentiation we utilized PC12 cells, a well-established cell model to address biological questions regarding this subject (also presented in various publications throughout this manuscript).

Starting from an ultrastructural characterization of the cell/nanostructure interface, we followed intracellular physiological conditions (i.e. FA dynamics, cytoskeletal organization, nanomechanical properties, CREB phosphorylation) that eventually influence and alter the cellular program and activities. Taken together, our data enable a causal link of the nanoscale topography-induced mechanotransductive events and emphasize the significant potential of nanostructured cluster-assembled substrates in influencing essential cellular functions, in particular in inducing (neuronal) differentiation processes. This potential could be utilized e.g. for the rational design of enhanced neural interfaces and circuits, devices promoting neurogenic events or tools for mechanotransduction studies.

## Results and discussion

### Cluster-assembled zirconia surface induces neuritogenesis

In a previous study we have shown that the nanoscale topographic cues of cluster-assembled titania surfaces produced by SCBD trigger neuritogenesis in the neuron-like PC12 cells in the absence of a biochemical stimulus [15]. An involvement of processes associated with cell adhesion and the cytoskeleton was broached but the detailed molecular mechanisms were not investigated.

**Fig. 1:**
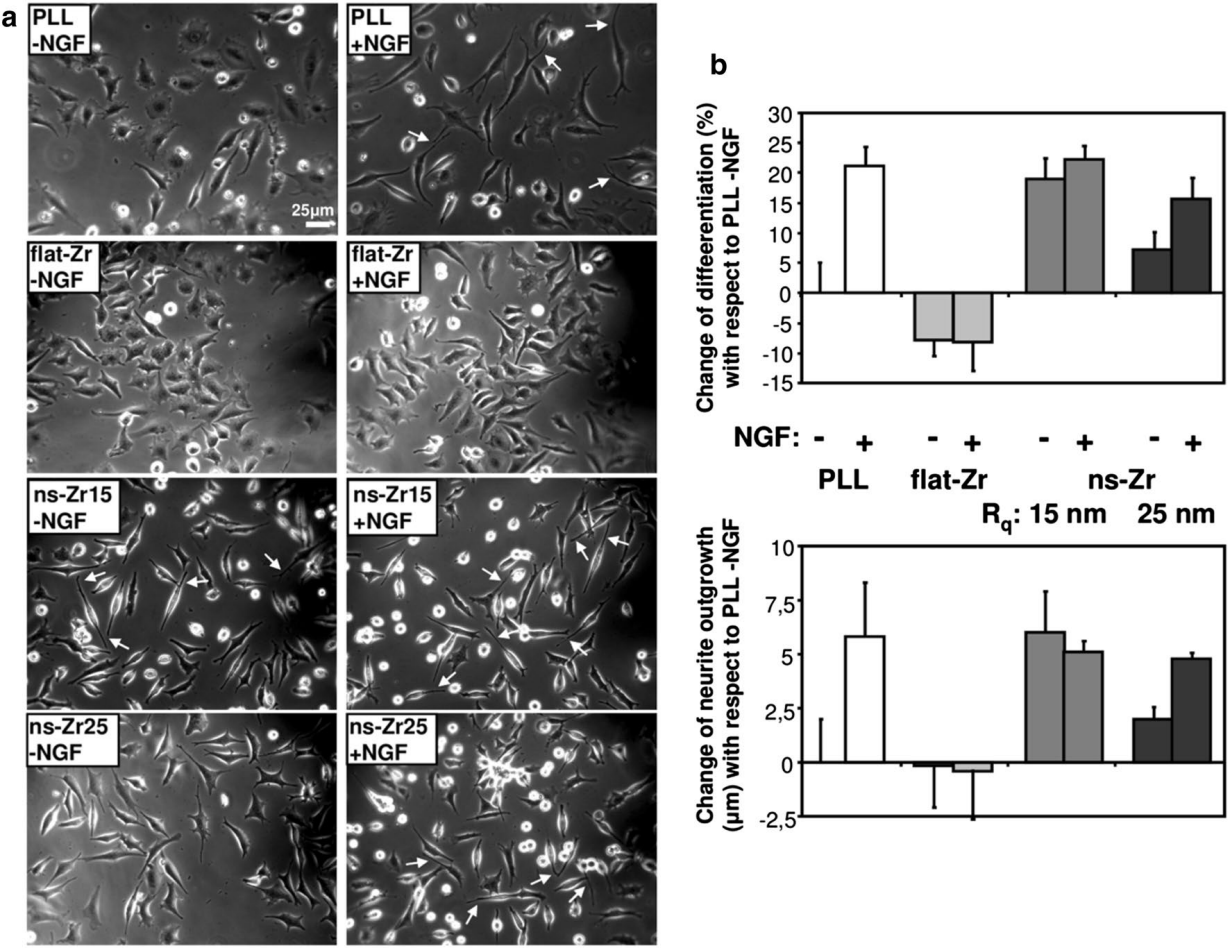
SCBD-produced nanostructured zirconia induces neuronal differentiation in PC12 cells. **a** The phase contrast images demonstrate the biological responses of neuron-like PC12 cells after 24 h interaction with the different surfaces presented in Fig. 2 in the absence or presence of nerve growth factor (NGF). *White arrows* indicated typical examples of neurite outgrowth of differentiated PC12 cells (in Additional file 1: Figure S1, **a** close up image of representative differentiated cells on ns-Zr15 is shown to illustrate more detailed the features of differentiated PC12 cells). **b** On the right the corresponding statistical quantification of the differentiation rate (*top*) and neurite outgrowth (*bottom*) is shown. A cell that developed at least one neurite with a length >10 μm was counted as differentiated, the quantification of neurite outgrowth are detailed in the “Methods” section. The *bars* represent the change of differentiation and neurite outgrowth compared to the PLL condition in the absence of NGF. The *bars* represent the average and are shown with the SD, representing the global statistics of five independent experiments (n: >500 cells, >150 neurites)

This phenomenon occurs also for cluster-assembled zirconia substrates with different nanoscale roughnesses. Figure 1a displays the biological response of PC12 cells after 24 h of interaction with different substrates, including flat zirconia (flat-Zr), nanostructured cluster-assembled surfaces with different roughness parameter R_q_ of 15 nm (ns-Zr15) and 25 nm (ns-Zr25), and as canonical control poly-l-lysine (PLL)-coated glass, with and without the nerve growth factor (NGF). As criterion for differentiation the presence of at least one neurite (cell projections growing from the soma of a neuronal cell during the initial phase of neuronal differentiation) with a length > 10 μm was imposed (some prominent examples are indicated with arrows in Fig. 1a and in a close-up in Additional file 1: Figure S1 the features of differentiated PC12 are illustrated).

Nanostructured zirconia induced differentiation and therewith neuritogenesis even in the absence of NGF, with the strongest effect on ns-Zr15 surfaces. Here, the differentiation and neurite outgrowth was in the range of the canonical condition achieved by NGF stimulation of PC12 cells plated on PLL (Fig. 1b). Also the rougher ns-Zr25 surfaces triggered differentiation, yet to a lower extent, which could be complemented, though, by the addition of NGF. Cells on flat-Zr surfaces instead did not show any sign of neuritogenesis, not even if they were exposed to the NGF stimulus (Fig. 1b). The potential of zirconia surfaces to induce NGF-independent neuritogenesis are thus correlated to their nanoscale morphological properties.

### Characterization of surface nanoscale morphology of cluster-assembled ZrO_2_ films

**Fig. 2:**
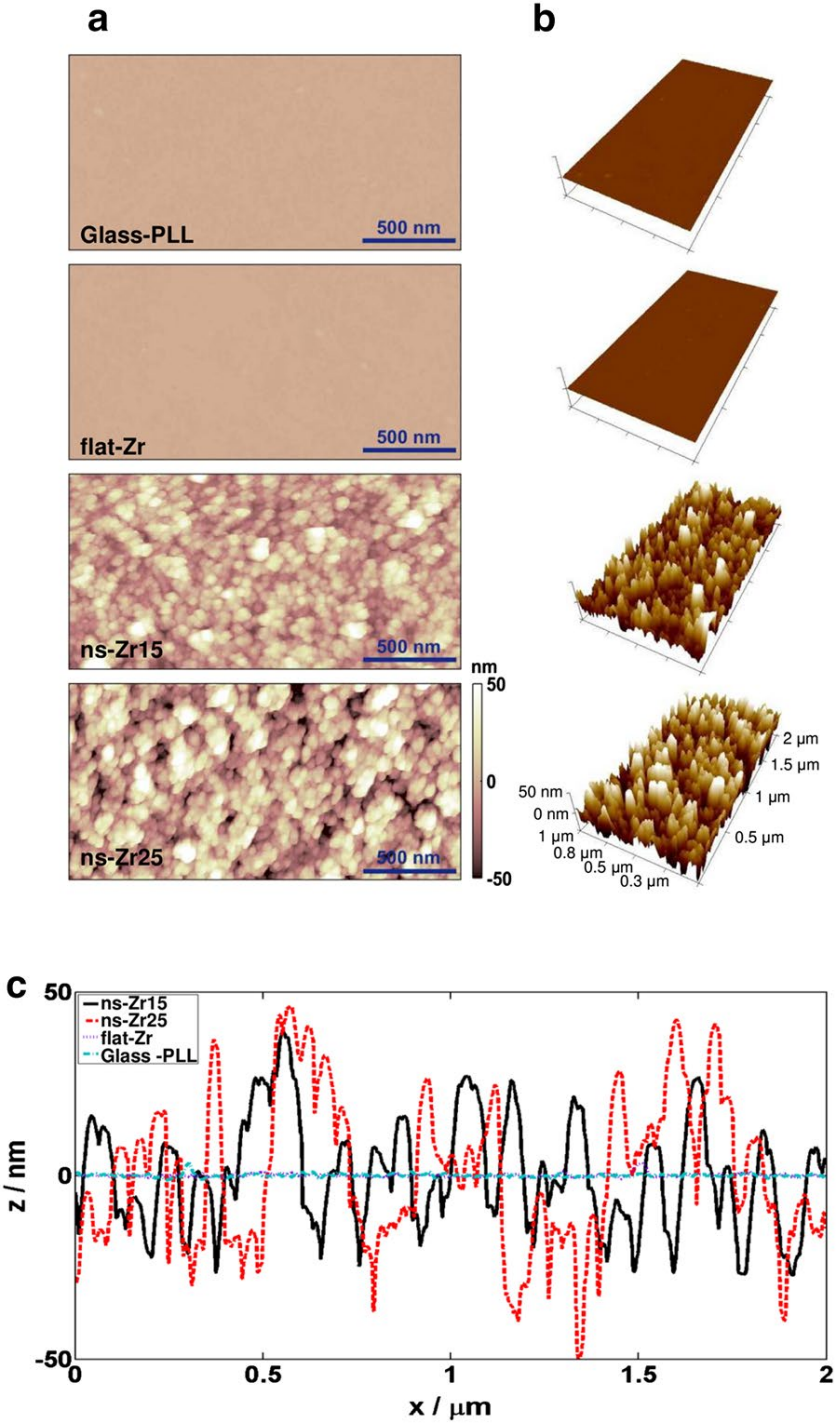
AFM morphological analysis of control and nanostructured surfaces produced by SCBD. The images show representative **a** *top views* and **b** *3-dimensional views* of the surfaces morphology of glass coated with poly-l-lysine (PLL), flat zirconia (flat-Zr) produced by e-beam evaporation, and nanostructured zirconia (ns-Zr) produced by SCBD with R_q_ = 15 (ns-Zr15), or 25 nm (ns-Zr25), respectively. **c** The *graphs* display a comparison of representative topographic profiles of different substrates

Figure 2a, b show typical AFM topographic maps (Fig. 2a: top- and Fig. 2b: 3-dimensional views) of PLL-coated glass, flat-Zr, ns-Zr15 and ns-Zr25 surfaces. PLL-coated glass and flat-Zr are very smooth (R_q_ < 1 nm) compared to the nanostructured ZrO_2_ films of different nanoscale roughnesses, as evident from the comparison of representative surface profiles shown in Fig. 2c.

The surface profiles of cluster-assembled zirconia films show peaks and valleys defining complex random patterns with features whose size and spatial distribution resemble those of the ECM [7]. The structure and morphology of cluster-assembled films are characterized by the random hierarchical self-organization of nanometer-sized building blocks (the clusters) in larger and larger units (statistical scale invariance). This is substantially different from the highly regularly patterned nano- and micro-fabricated surfaces (i.e. pillars, gratings, holes) usually employed in the vast majority of nanotopography-related studies of biomaterials [2, 4, 12]. Although the presence of topographic disorder at the nanoscale has been shown to have a large influence on cell adhesion, integrin clustering and differentiation [2, 24], no systematic characterization of the influence of disordered substrates with different nanoscale features has been reported so far.

The complexity of the cluster-assembled zirconia morphology is the result of the growth mechanism of interfaces produced under the ballistic deposition regime (BDR), this regime is typical of the SCBD technique [23, 25]. In BDR, elemental building blocks (atom clusters in our case) produced in the gas phase land on a substrate sticking without significant mobility; fragmentation of the clusters upon landing is inhibited due to their low kinetic energy [22]. In the framework of BDR regime, the nanoscale roughness of cluster-assembled surfaces can be quantitatively analysed and precisely reproduced, since it depends on simple scaling laws [23, 25]. This means that the topographical features and the evolution of disordered cluster-assembled substrates can be described by simple mathematical models [25]. In particular, the surface roughness R_q_ and other morphological parameters (e.g. specific area, slope, lateral correlation length) in the BDR regime depend on the film thickness and typically increase with it [23]. By carefully characterizing the evolution of the nanoscale surface parameters with film thickness it is thus possible to obtain a calibration allowing the precise and reproducible control over the surface morphology evolution by controlling the SCBD deposition parameters [23].

### Potential of nanostructured zirconia surfaces in modulating cell adhesion-related processes

Nanoscale roughness is an important parameter influencing the interaction of surfaces with proteins and cells [2, 4, 7, 26, 27], however, it does not provide details about the precise surface nanoscale information relevant for the cell and hidden in the configuration of the random layout of asperities. Yet, it is this configuration which is likely to impact on mechanotransductive processes in cells via the modulation of cell adhesion-related processes, in particular regarding integrin clustering and FA maturation/composition [2, 4, 24].

It was therefore important to study the asperity layout to understand profoundly what kind of nanoscale information is potentially perceived by cells interacting with the cluster-assembled zirconia surfaces. As a first step, we visualized and analyzed the actual cell/substrate interface. We achieved the visualization via ultrathin section images of the interaction interface recorded by transmission electron microscopy (TEM).

**Fig. 3:**
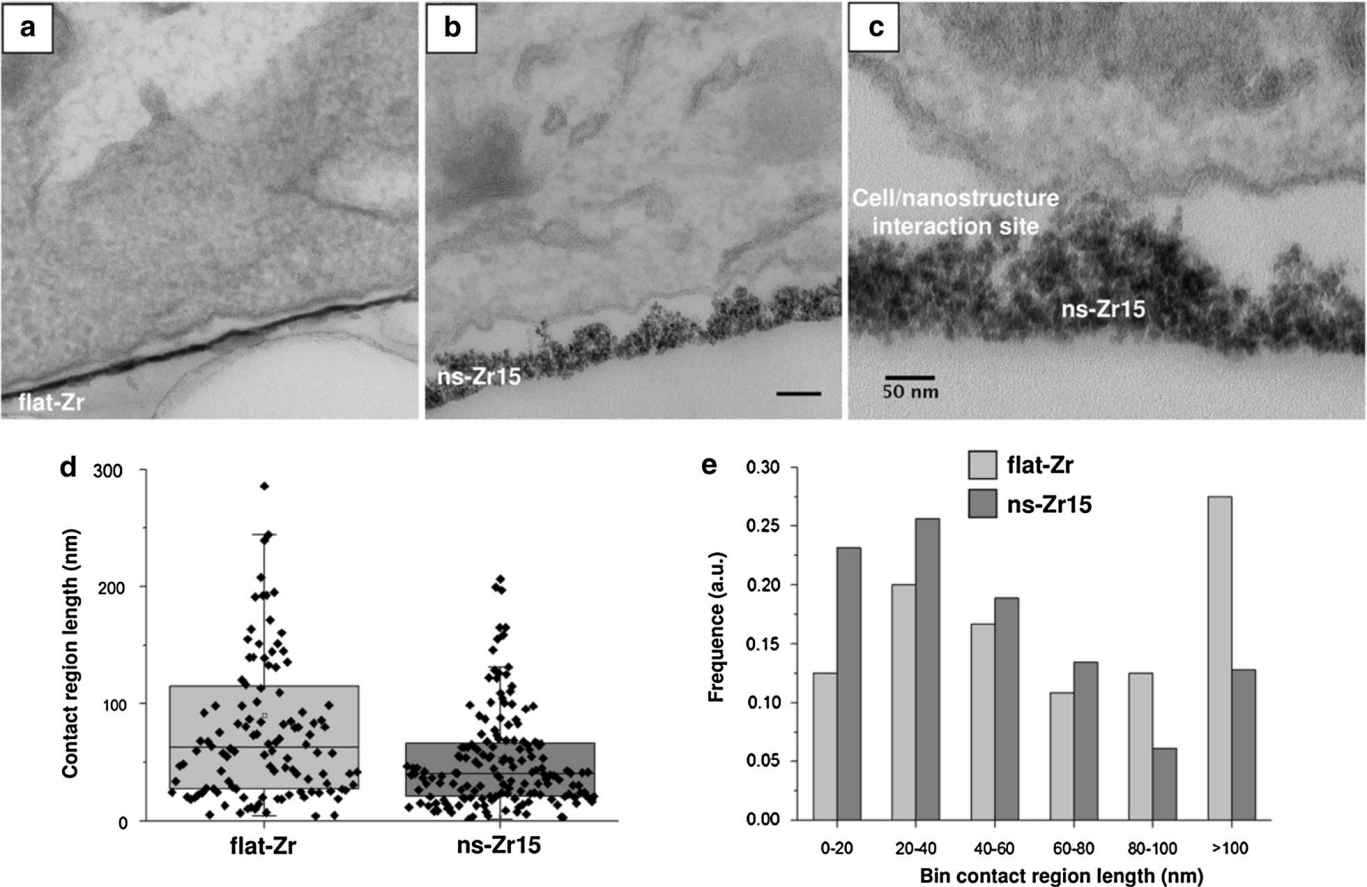
Nanostructured zirconia surfaces alter the nanoscale adhesion site architecture. Representative TEM images of the cell/substrate interface on **a** flat-Zr, and **b** ns-Zr15 substrates. The* scale bars* equal 100 nm. **c** This image shows a close-up of a representative interaction zone between a PC12 cell and the ns-Zr15 surface. The cells interacted with the surfaces for 24 h. A description of experimental setting and quantification can be found in the “Methods” section. In d, e the corresponding analysis of the width of the adhesion sites at the nanoscale are summarized. **d** The absolute values of all measurements for both substrates are shown. **e** The *histogram* of measured widths of adhesion sites are displayed here. The *graphs* represent the global statistics of images obtained from two independent experiments (fl-Zr: n = 120, ns-Zr15 = 164)

An exemplary illustration of the cellular interaction with flat-Zr and ns-Zr15 substrates is shown in Fig. 3a–c. A first important observation is that the cell membrane does not follow strictly the topographical profile of the surfaces; the cell instead interacts with the substrate at isolated locations, separated by regions where contact is not established. Figure 3b, and in particular the close-up in Fig. 3c, show that contact between the cell and the nanostructured substrate is achieved in correspondence of the apical part of the most protruding surface asperities. From the TEM sections contact regions have been quantitatively characterized and the results are reported in Table 1; Fig. 3d,e. While the spatial occurrence of contact regions (the average distance between nanoscopic adhesion regions) is statistically similar on the two surfaces, contact regions on flat-Zr (average diameter: 90 nm) are highly significantly (p value = 0.0002, Wilcoxon–Mann–Whitney test) larger than those on ns-Zr15 (average diameter: 53 nm) (Table 1).

**Table 1:** Characterization of the contact regions from TEM analysis

	Flat-Zr	ns-Zr15
Width of contact regions (nm)	Av.: 90.2 ± 93.2	Av.: 53.2 ± 48.0
	(SD) ± 16.9 (95 % CI)	(SD) ± 7.4 (95 % CI)
	Median: 62.8 ± 37.5	Median: 40.4 ± 21.6
Distance between contact regions (nm)	Av.: 108.6 ± 101.9	Av.: 99.1 ± 101.4
	(SD) ± 21.9 (95 % CI)	(SD) ± 18.6 (95 % CI)
	Median: 73.7 ± 38.6	Median: 60.4 ± 33.4

**Fig. 4:**
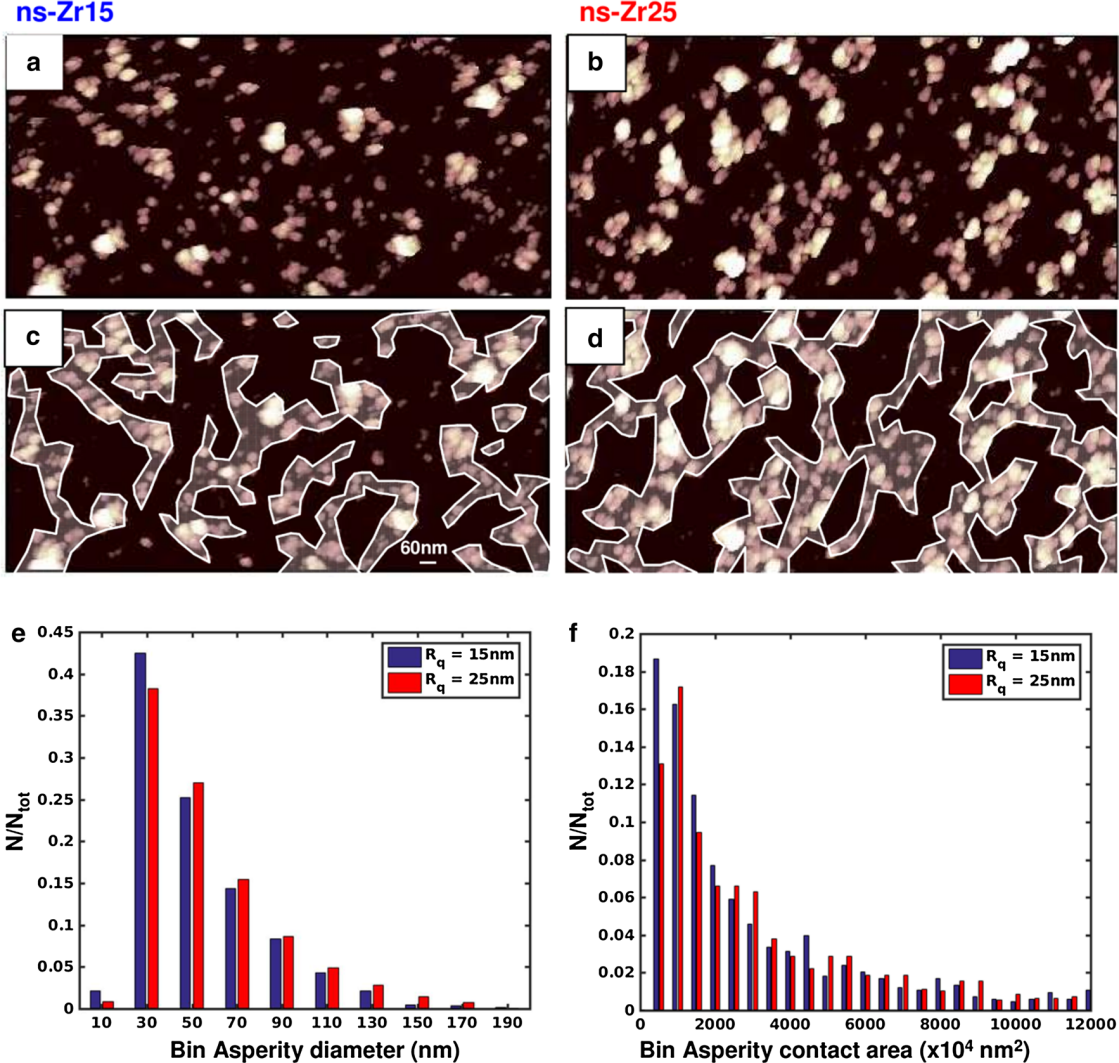
SCBD-produced nanostructured zirconia surfaces have the potential to modulate adhesion-related processes. **a, b** Representative asperity maps of ns-Zr substrates with different roughness (R_q_ = 15, 25 nm, respectively). For details, see Additional file 2: Figure S2 and “Methods” section. **c, d** Asperity clusters highlighted from the asperity maps shown in panels **a, b** after grouping asperities separated by less than 60 nm (a reference *bar* with length 60 nm is shown). **e, f** The* graphs* show cumulative distribution of (**e**) asperity diameters and (**f**) the asperity area of ns-Zr15 and ns-Zr25 substrates

Based on this initial observation, we determined from AFM topographic maps the characteristics of the potential individual nanoscopic contact sites provided to the cells by the nanostructured surface, which represent the actual nanoscale information sensed by the cells (for details on the approach, see “Methods” section and Additional file 2: Figure S2). Figure 4a, b shows representative asperity maps for ns-Zr15 and ns-Zr25 surfaces and Table 2 reports the median values and median absolute deviations (MADs) of the relevant parameters of surface asperities. The distributions of asperity diameters and contact areas are shown in Fig. 4e, f. The asperity diameters and the potential contact area for cells provided by the individual asperities are significantly smaller for the ns-Zr15 sample (diameter: −9 % but still significant with p value = 0.01, as the sample size (>2000) is large, area: −25 % p value = 0.001, double-sided t-test). Remarkably, the distributions of surface asperity diameters determined by AFM (Fig. 4e) and of adhesion contact widths from TEM sections (Fig. 3e) are quite similar (median values are 43 and 40 nm, respectively).

**Table 2:** Characterization of the contact asperities from AFM analysis

	ns-Zr15	ns-Zr25
Distance between asperities (nm)	72 ± 8	87 ± 10
Asperity diameter (nm)	43 ± 15	47 ± 17
Asperity contact area (nm^2^)	1954 ± 1268	2596 ± 1766

Together with intrinsic cellular conditions (discussed in detail in the following section “Intracellular processes induced by the cellular interaction with the nanoscale roughness of zirconia substrates: focal adhesion dynamics and cytoskeletal organization”), there are several pivotal features of extracellular microenvironment configuration which are decisive for the formation of superior adhesion structures, such as nanoclusters or even FC/As realized by integrin clustering and adhesome complex assembly [2, 3].

One of them is the available nanoscale adhesion area as there seems to exist a minimal adhesion unit (>4–9 integrins) [2, 28], but considering the size of integrins (~15–20 nm in height and width of the extracellular domain) [29], most of the individual asperities offer large enough contact area dimensions to establish this minimal adhesion unit.

Moreover, critical ligand spacing thresholds between adhesion sites have been identified to influence the cellular capability to establish FAs [1, 2, 30], force transmission and mechanoregulation [31]. Interestingly, with a value of ~60–70 nm these thresholds are in the range of what was determined as the median of the asperity separation of our tested surfaces.

To get a visual prediction of how the asperity layout and the distances between the asperities might impact on integrin clustering and FA formation, we have grouped neighboring asperities applying a spacing threshold of 60 nm. This procedure enabled us to define potential asperity clusters which might permit the intracellular formation of the aforementioned superior adhesion structures (Fig. 4c, d). It can be recognized that the ns-Zr surface features set spatial constraints for the asperity clusters, in a manner that they remain mostly of small dimensions, actually in the range of nanoclusters or FCs [32]. For further maturation of the nascent adhesions into FCs or even into FAs, though, the surface characteristics might be quite restrictive. This is true in particular for ns-Zr15, whereas for ns-Zr25 these effects are mitigated because the asperity clusters are of notably larger dimensions (compare Fig. 4c,d), probably also as a consequence of the larger contact area and diameter of single asperities (Fig. 4f). On glass-PLL or flat-Zr apparently no such substrate-induced topological constraints exist.

Summarizing, our analysis suggests that nanostructured surfaces produced by SCBD possess a significant potential in modulating cell adhesion-related processes, e.g. by restricting integrin clustering and FA maturation [2, 4], and that the conditions for expressing this potential are better satisfied in the case of ns-Zr15, the sample with lower roughness. In the proceedings of our study we have therefore focused our attention only on ns-Zr15 samples, which also induced the strongest biological effect; using flat-Zr and the canonical PLL-coated glass surfaces (±NGF) as references.

### Intracellular processes induced by the cellular interaction with the nanoscale roughness of zirconia substrates: focal adhesion dynamics and cytoskeletal organization

The analysis of the cell/substrate interface predicted hence a possible impact of the nanoscale roughness on integrin clustering and FA formation, which prompts a deeper examination of these processes and the receptors and signaling cascades effectively involved in mediating the observed morphological effects.

**Fig. 5:**
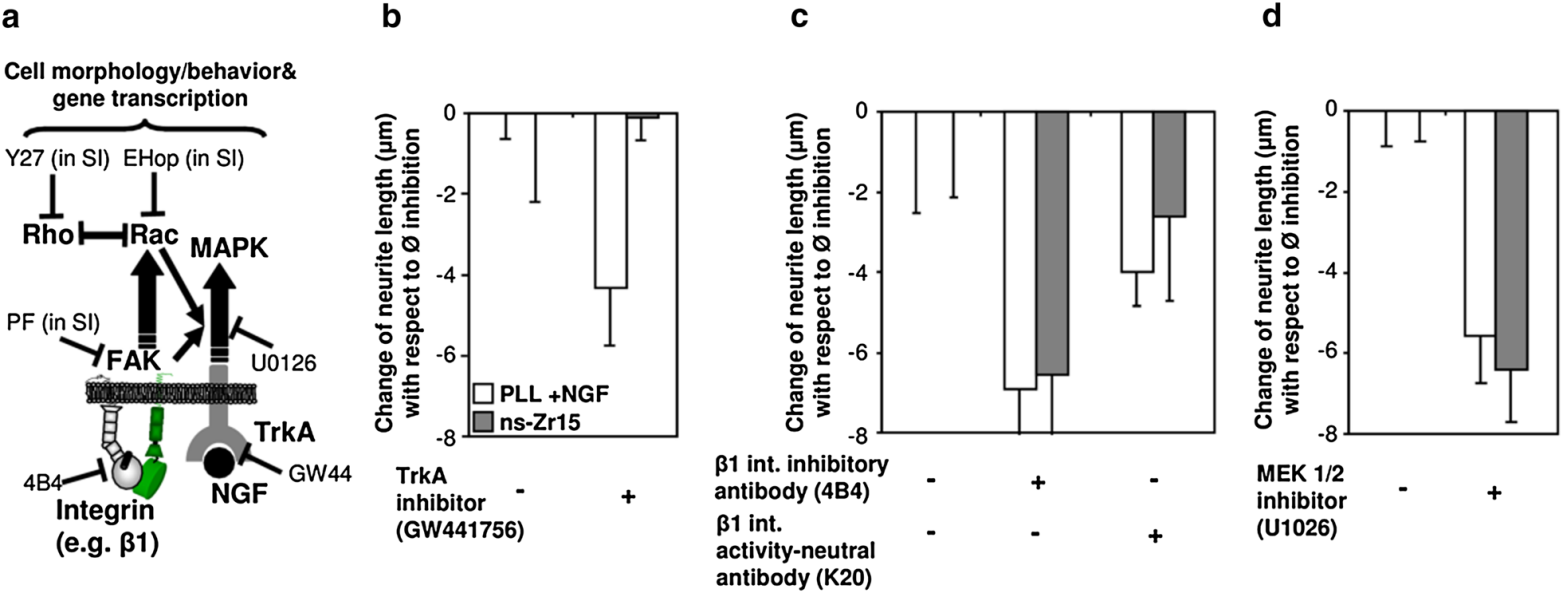
NGF and TrkA activation are dispensable in nanostructure-induced neuritogenesis whereas β1 integrin activation/signaling are essential. **a** The *scheme* illustrates the interference points of the various inhibitors of proteins involved in the integrin and RTK signaling cascade which were used in the experiments. **b–d** The PC12 cells were plated on PLL (+NGF) or surfaces with a roughness R_q_ of 15 nm rms. In case of inhibitor treatment, the inhibitors were preincubated for 15 min prior to cell plating and then present for the whole ongoing experimental period. The cell morphology was recorded by phase contrast microscopy 24 h after plating the cells. As biological read-out for the differentiation the quantification of the neurite outgrowth is shown (obtained with the help of ImageJ); representative images can be found in Additional file 1: Figure S1. The *bars* display the change of neurite outgrowth compared to the situation without treatment on the corresponding substrate. The *bars* are flanked by SD. The *graph* displays the results of an inhibitor treatment against **b** TrkA (GW441756 1 µM, from two independent experiments), **c** the incubation with the 4B4 inhibitory antibody (2.5 µg/ml) (or activity-neutral antibody K20 (2.5 µg/ml) as control) against β1 integrin (from three independent experiments) or **d** the inhibition of MEK 1/2 (U1026 10 µM, from two independent experiments), always both in the canonical (NGF-induced) and the nanostructure-induced condition (n: >500 cells, >150 neurites). Further inhibitions of major mediators and processes involved in integrin signaling and cytoskeletal organization are displayed in Additional file 3: Figure S3

The canonical neuritogenesis in PC12 is typically activated by binding of NGF to TrkA (which represents the principal NGF receptor of these cells) [33] and predominantly mediated via the MAPK/Erk signaling cascade [34] (Fig. 5a). Consequently, the inhibitor GW441756 (a selective inhibitor of TrkA) diminished this kind of NGF-induced differentiation, whereas it was interesting to notice that it was ineffective for the nanostructure-induced differentiation (Fig. 5b), thus excluding that the nanotopography activates TrkA.

As aforementioned, the receptor family of integrins plays an eminent and essential role in FA-mediated cell adhesion and also (in particular β1 subunit-containing integrins) in the regulation of neuronal differentiation, neurite/axon outgrowth and pathfinding [35–37]. An involvement of FA-related processes in nanotopography-promoted cellular differentiation is likely [2, 4, 9] and has been addressed recently by Yang et al. in a neuronal context [20], but nevertheless many aspects are still unclear [2, 4, 9, 10]. In contrast to the TrkA inhibition, an allosteric inhibitor of β1 integrin activity (antibody 4B4) strongly impaired the growth of neurites in both conditions (Fig. 5c). K20, a β1 integrin-binding, but activity-neutral antibody instead interfered only moderately (Fig. 5c).

Moreover, also the inhibition of further prominent mediators and structural compoments in the integrin/FA signaling cascade (lipid rafts, FAK, RhoGTPases, cytoskeleton, actomyosin) confirmed the involvement of this signaling pathway (Additional file 3: Figure S3A, B). Interestingly, despite the dispensability of NGF (Fig. 1) and TrkA activation (Fig. 5b), the induction of the MAPK pathway was necessary also in nanostructure-induced neuritogenesis as inhibition experiments with U-0126 (an inhibitor against MEK1/2) demonstrated (Fig. 5d). In fact, it is well-known that the integrin and MAPK signaling pathways are interlaced [38] (Fig. 5a) and that MAPK pathway activation by β1 integrin plays an important role in neural stem cells [35].

Our results show that NGF and TrkA activation are not required for the nanostructure-induced PC12 differentiation. However, the same biomechanical, cytoskeletal and FA-related structural and signaling components are essential to realize the outgrowth of neurites, independent of whether the given neuritogenesis-inducing stimulus is NGF or triggered by the cellular interaction with a nanostructure providing the adequate roughness.

**Fig. 6:**
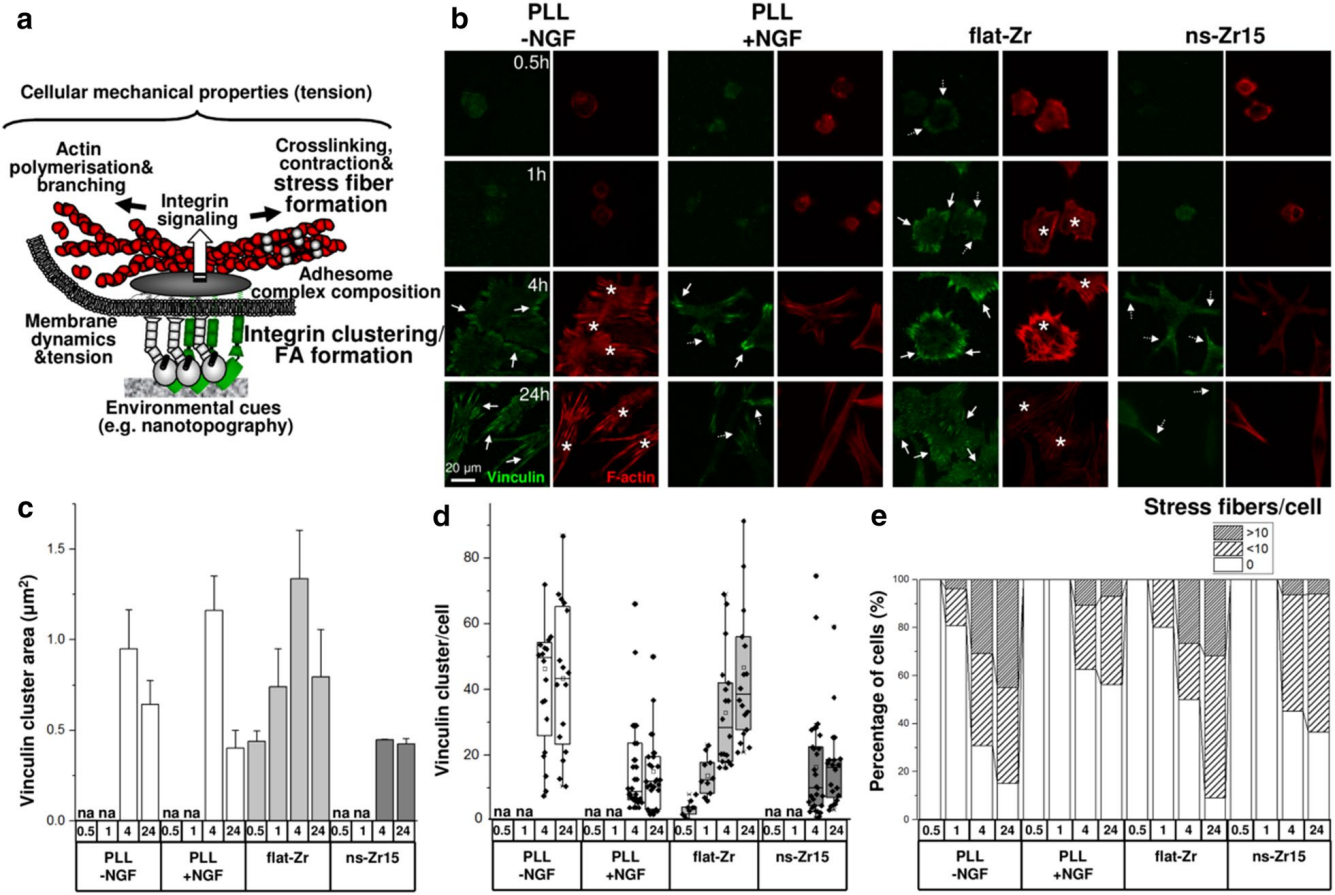
Focal adhesion formation/dynamics and cytoskeletal organisation differ on flat or neuritogenesis-inducing nanostructured surfaces. **a** The* graphic* visualizes and summarizes the cytoskeletal and mechanobiological processes which are influenced by FA organization and dynamics. **b** In the* panel* representative images of the PC12 cells fixed with 4 % PFA after the indicated time periods on the different surfaces are demonstrated (vinculin staining recorded by TIRF microscopy, f-actin in epifluorescence). The *white arrows* indicate exemplary areas with focal complex (*pointed line*) or focal adhesion (*continuous line*) structures. The* asterisks* pinpoint to exemplary areas with strong actin fiber formation. **c**–**e** The *graphs* summarize the corresponding results (representing the global statistics of three independent experiments, vinculin clusters: n = 722–3678, cells: n = 16–34) of the quantifications (obtained with ImageJ) of **c, d** FA dynamics; **c** Vinculin cluster area, **d** Number of vinculin clusters per cell, and **e** cytoskeletal actin fiber organization. *na* not analyzable

Apart from this general involvement of the same structural and cell adhesion signaling components, the combined TEM and AFM analysis (Fig. 3, respectively 4) suggested that differences in FA formation might arise between the flat and nanostructured surfaces. In particular for the neuritogenesis-inducing ns-Zr15 surface a partially frustrated potential in permitting the formation of superior adhesion structures, especially mature FA, was predicted (Fig. 4c, d). We have discussed in the precedent section “Potential of nanostructured zirconia surfaces in modulating cell adhesion-related processes” that FA formation depends on extracellular microenvironmental features such as area, geometry, ligand spacing and nanotopography of the adhesion sites [1, 2, 4, 30, 39]. Intracellularly, FA signal transduction is regulated by the nanoscale architecture/composition of the adhesion complex, the force and tension development between integrins and the actomyosin network [2, 3, 10, 32, 40–42] (Fig. 6a). However, the exact mechanism of this cellular environmental (mechano) sensing and especially its impact on differentiation processes is intricate and still only partially understood [1–4, 6, 10, 11].

We therefore investigated the impact of cellular interaction with the different surfaces on the spatiotemporal dynamics of FA formation. Vinculin staining at different time points recorded by TIRF microscopy confirmed that the FA dynamics and the extent of their maturation clearly vary between flat and nanostructured surfaces (Fig. 6b–d). In the canonical condition on glass-PLL we observed an evident formation of small FC-like structures and also more mature FAs after a few hours. Without NGF the presence of these structures remained high, whereas in the case of NGF stimulus they reduced in number towards the 24 h time point and became smaller. On flat-Zr the FC/A formation was accelerated and clearly enhanced. Already after half an hour apparent accumulations of vinculin were detectable. After 1 h and later on distinct and numerous FAs were detectable, mainly at the cell border.

On ns-Zr15 substrates the situation was instead quite different. Diffuse punctate staining, indicating nanoclusters or FCs, was visible starting from the 4 h time point but FAs basically did not form, not even at later time points (Fig. 6b–d). For the correlated formation of high order actin filament structures (e.g. stress fibers) the situation was similar. They were mainly established on the flat substrates Glass-PLL and flat-Zr, but to a much lower extent on the neuritogenesis-inducing ns-Zr15 (Fig. 6e).

We demonstrated the indispensability of β1 integrin activation and the alteration of the FA configuration, but it remained unclear whether direct integrin/nanostructure interaction is taking place. It can be speculated, though, that the interaction is mediated with involvement of adhesome complexes. Moreover, it has been shown recently that cell adhesion mediated by non-integrin anchoring receptors can trigger integrin activation/signaling via membrane tension also independently of actual integrin/ligand binding [43].

We further investigated whether this modulation of the FAs and the cytoskeleton was causally involved in the nanostructure-driven induction of neuritogenesis. To contrast the altered FA dynamics and cytoskeletal organization on ns-Zr15 we chose to treat cells with lysophosphatic acid (LPA). LPA is long-known for its ability to remodel the actin cytoskeleton by inducing Rho signaling-dependent formation of FA and stress fibers, and to cause therefore subsequently neurite retraction in PC12 [44]. Congruently, the treatment reduced both, the NGF- or nanostructure-induced neurite outgrowth, at higher LPA concentration. In the nanostructure-induced neuronal differentiation, though, already lower concentrations of LPA had an inhibiting impact on the neurite outgrowth (Additional file 3: Figure S3C).

Neuritogenesis is a complex case of cellular morphogenesis. Neurite budding requires a concerted interplay between the neuronal actin and tubulin cytoskeleton components in order to break the neuronal sphere [45]. Furthermore, once a neurite is initiated the processes driving neuritogenesis and in particular the growth cone advancement [36] are very similar to the ones in mesenchymal cell migration and depend therefore on a highly regulated crosstalk between Rac1 and RhoA orchestrating the turnover of contact points [36, 46] and mediating thereby an appropriate, balanced ECM/integrin/actin cytoskeleton linkage [36, 41, 42, 47–49] and force generation by the molecular clutch [11, 41, 50, 51]. In fact, growth cones itself have been determined as rather soft cellular structures [50] requiring small point contacts with a dynamic turnover for efficient motility [36, 52]. It can be speculated that on flat-Zr the adhesion might be utterly enhanced to a point that leads to more stable/less dynamic focal adhesions imposing an overall anti-differentiation biomechanical condition, which makes it difficult to break the neuronal sphere in the initial phase of neurite budding and/or to promote efficiently the growth cone advancement, thereby even contrasting the NGF stimulus. On the contrary, ns-Zr15 surfaces seem to set an ideal cellular status of adhesion and cytoskeletal organization to favor neuritogenesis, even in the absence of a biochemical stimulus. Further experiments are necessary to eventually elucidate these aspects.

However, together the presented complex of data on FA dynamics/signaling and the cytoskeleton propose a particular importance of the mechanotransductive aspect in the nanostructure-induced condition.

### Modulation of the cellular mechanical properties is the key signal integration for the nanostructure-induced neuronal differentiation processes

In fact, cell/substrate interactions, the integrin engagement to the actomyosin network and correlated FA signaling strongly impact on the organization and the biomechanical, tensional state of the cytoskeleton [3, 6, 41, 42]. Effectively, cellular mechanics have been suggested to be by themselves a signal integrator which potentially not only affect the cell morphology [53] but also essential cell functions and eventually the cell’s fate [5, 6, 10, 41] (Fig. 6a), in particular also in neuronal cells [11]. In mesenchymal stem cells it was hypothesized that effects on FAs, the cytoskeleton and cellular mechanics were provoked by adding nanogratings (350 or 500 nm width) to the substrate [54].

As described above, our experiments demonstrate that integrin signaling and cytoskeletal dynamics are essential for both canonical and nanostructure-induced neuritogenesis (Fig. 5; Additional file 3: Figure S3A, B). The data also accentuated, though, decisive differences in the nanoarchitecture of the cell/substrate interface (Figs. 3, 4), FA dynamics and actin filament organization (Fig. 6b–e) between cells on flat-Zr and neuritogenesis-inducing ns-Zr15 surfaces. Therefore it was important to understand whether different cell/substrate interactions might have an effect on the overall cellular mechanical properties.

**Fig. 7:**
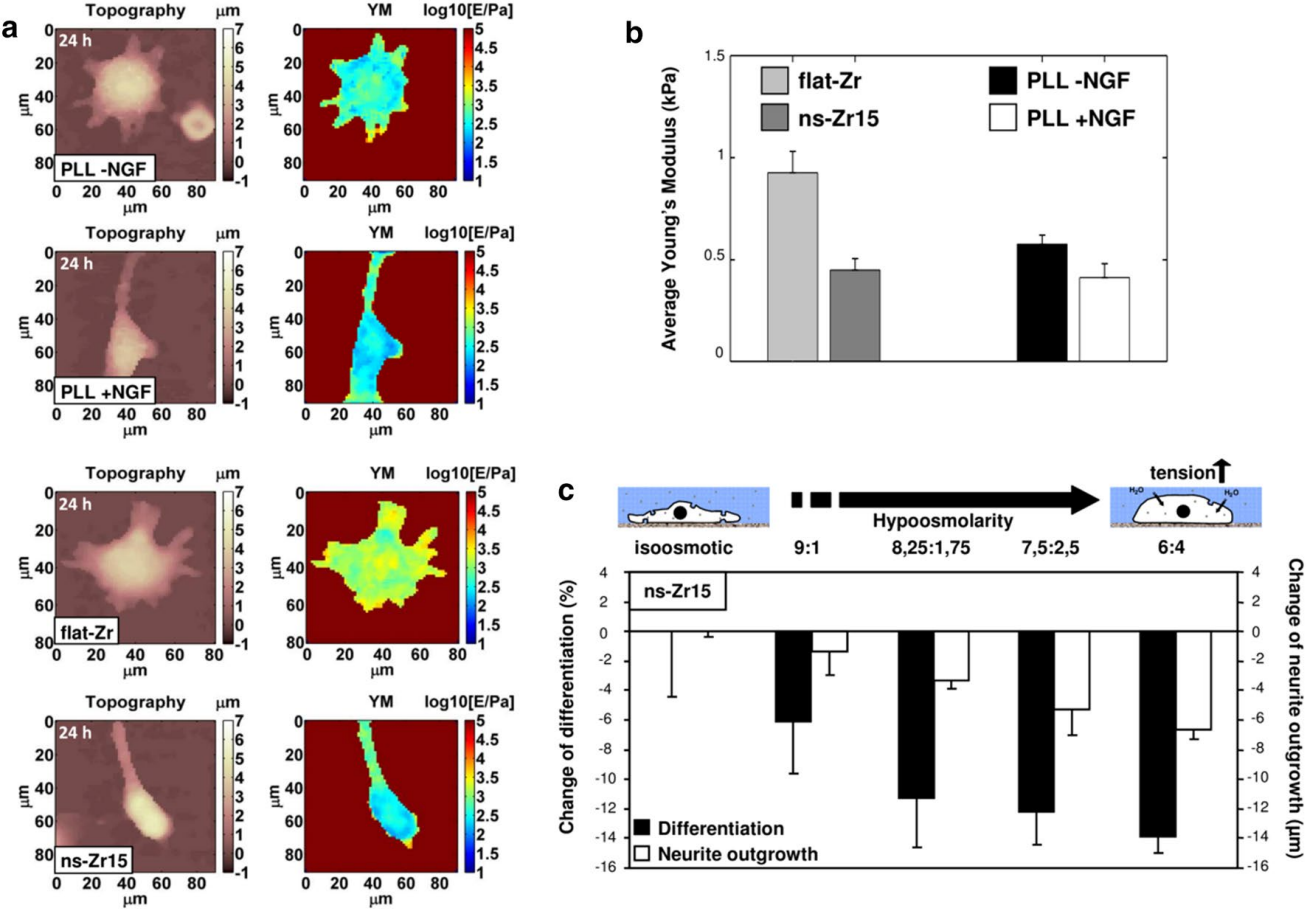
Cellular rigidity is decreased on the neuritogenesis-inducing surface, being the decisive signal for the differentiation. **a** Representative morphological images (*left images*) and maps of the Young’s modulus of elasticity (*right images*) of living PC12 cells interacting with PLL (in the presence or absence of NGF), or with flat or the nanostructured neuritogenesis-inducing zirconia surfaces. **b** On the *upper right*, the *graph* displays the summary of the corresponding analysis of the biomechanical properties of the membrane/cytoskeletal layer. The *bar* represents the average of the global statistics obtained from two (flat-Zr, Glass-PLL ±NGF), respectively three (ns-Zr15) independent experiments (number of measured cells: flat-Zr: n = 6, ns-Zr15: n = 7, PLL −NGF: n = 8; Glass-PLL +NGF: n = 8), flanked with the error which was calculated as described in the “Methods” section. YM young’s modulus. **c** Differentiation rate and neurite outgrowth of PC12 cells plated for 24 h on the neuritogenesis-inducing ns-Zr15 surface in the presence of isoosmotic medium or in medium with the indicated hypoosmolarity. The *bars* represent the average of two independent experiments and are flanked with the SD (n: >500 cells, >150 neurites). Representative images of all conditions can be found in Additional file 3: Figure S3

We performed AFM-based nanomechanical measurements of living PC12 interacting with PLL ±NGF, flat-Zr and ns-Zr15 surfaces (Fig. 7). Interestingly, in the latter case the membrane/cytoskeletal layer of the somas of the cells were characterized by a highly significantly lower rigidity (−51 % in the Young’s modulus, p value < 0.01, double-sided t test) compared to the cells on flat-Zr (Fig. 7b). Similarly, in the cells on glass-PLL a decrease of the rigidity was notable between the undifferentiated cells without NGF stimulus and the differentiated cells in the presence of NGF (−28 % in Young’s modulus, p value < 0.1), arriving at a level comparable to the one of the cells on ns-Zr15 (Fig. 7b). The fact that the cells in the flat-Zr condition demonstrate the highest absolute rigidity value of all tested conditions (p value PLL −NGF vs flat-Zr < 0.02) is furthermore congruent with the aspects of enhanced cell adhesion and neuritogenesis-unfavorable biomechanical state in this condition, discussed in the section “Intracellular processes induced by the cellular interaction with the nanoscale roughness of zirconia substrates: focal adhesion dynamics and cytoskeletal organization”.

Taken together, the data strongly suggest that the status of the FA architecture/dynamics and the cytoskeletal organization—enforced by the specific characteristics of the neuritogenesis-inducing substrate—might be the reason for the decrease of the rigidity/tension in the somal membrane/cytoskeletal layer.

Keeping in mind the hypothesis of the mechanotransduction concept, these results on the impact of the ns-Zr15 surface on cellular FA formation, cytoskeletal organization and nanomechanics evoked the question concerning the role of the causal signal integration as the effective driving force of the nanostructure-induced neuronal differentiation.

To address this issue, we compensated the reduced rigidity/tension of cells plated on the neuritogenesis-inducing surfaces by hypoosmotic swelling. We observed that upon increasing of hypoosmolarity, the nanostructure-induced effects gradually decreased (Fig. 7c). Congruently, low hyperosmolarity even slightly increased the percentage of differentiated PC12. Higher hyperosmolarity levels had a slightly decreasing effect on differentiation due to general rounding up of the cells, but neurite length was basically unaffected (Additional file 3: Figure S3D).

We therefore conclude that the alteration of the cellular biomechanical properties—caused by the interaction with a neuritogenesis-inducing nanostructured surface and the subsequent induction of mechanotransductive events—is the decisive integrating signal permitting the promotion of neuronal differentiation. Altogether, to our best knowledge, this is the first robust evidence of a strong causal link between mechanotransductive processes and nanotopography-based biomaterial-induced biological effects.

### Dynamics of transcription factors controlling neurogenic processes induced by surface nanostructure

Our experiments demonstrate the pivotal significance of mechanotransductive signaling pathways for the nanostructure-induced neuronal differentiation. Typically signaling cascades are finalized by the activation of transcription factors (TF) and their binding to specific sequences of the DNA. Thereby they eventually realize the necessary change of the gene expression profile and cellular program.

CREB is a prominent TF which can be the endpoint of versatile signal inputs arriving from cAMP-, integrin-, RTK/MAPK/Erk-, NO-, calcium- or mechanical force-mediated signaling cascades or, very often, also a combination of them [55–57]. Consequently it functions as a signal integrator with a strong impact on the expression level of a wide range of genes [58], notably also in the control of early neurogenesis [56–58]. For these reasons CREB was a good first candidate in our context to examine downstream events on the level of transcription control relevant for early neuronal differentiation processes.

**Fig. 8:**
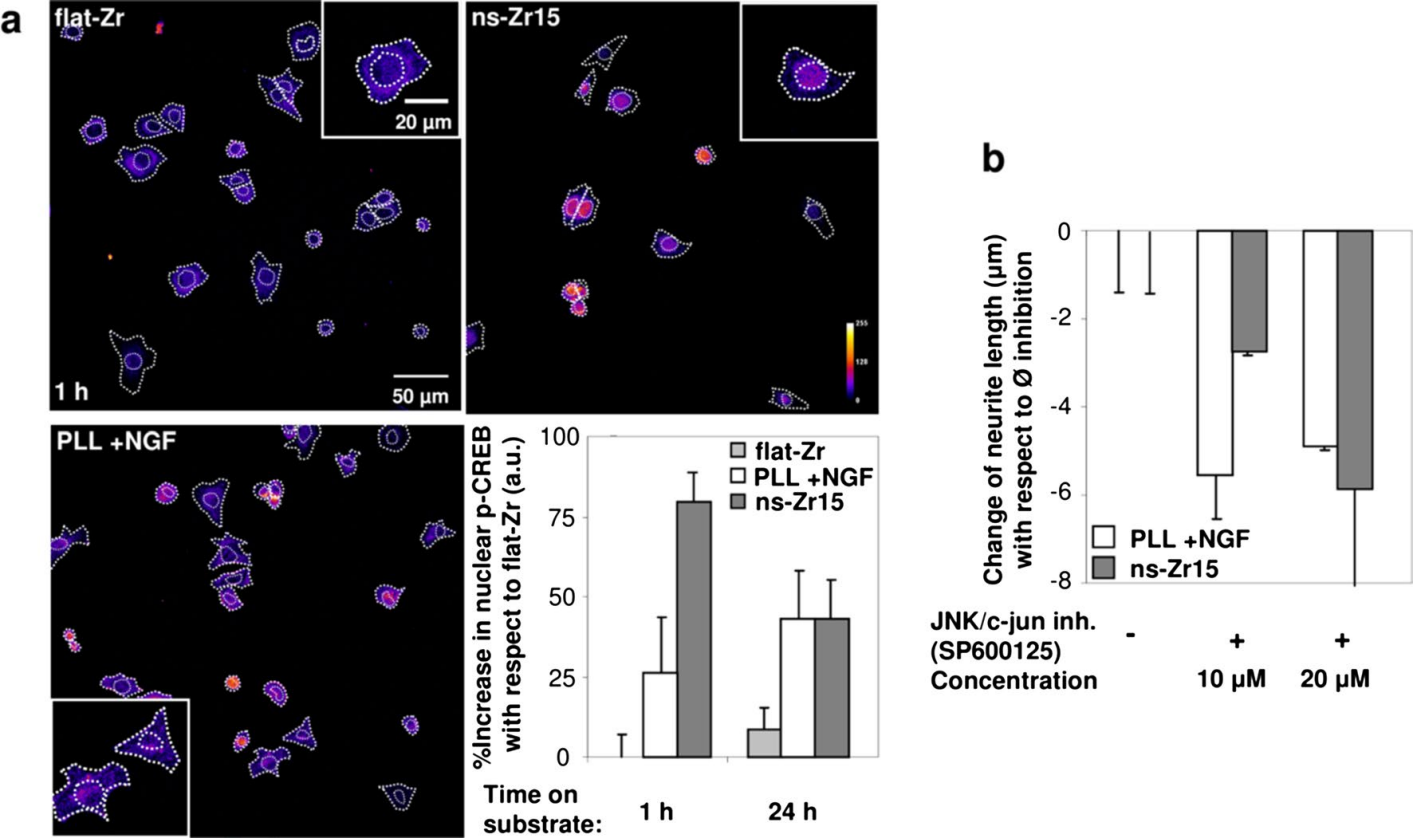
The interaction with the neuritogenesis-inducing surface has an impact on transcription factor dynamics relevant for neuronal differentiation. **a** The confocal images show the average stack projection of p-CREB stainings of cells in the indicated experimental conditions. The cells were fixed with 4 % PFA 60 min or 24 h after in the indicated conditions and stained for the nucleus (HOECHST), f-actin and p-CREB. The outer dashed lines represent the outlines of the cells obtained from the f-actin staining and the inner dashed line the nuclear area determined from the HOECHST staining. The* graph* (representing the global statistics of two independent experiments, n = 33–76 cells) summarizes the corresponding quantification performed with the help of ImageJ (see also “Methods”). **b** The same experimental procedure and quantification (from three independent experiments, n: > 500 cells, >150 neurites) as in Additional file 2: Figure S2A–C, but with an inhibitor against JNK/c-jun (SP600125 10 and 20 µM). Representative images of the conditions can be found in Additional file 3: Figure S3

We performed a confocal imaging analysis of nuclear CREB phosphorylation (which activates this TF) (Fig. 8a). On flat-Zr there was no detectable phosphorylation of the nuclear CREB. For the canonical NGF stimulation a slight, but significant nuclear p-CREB signal was visible after 1 h which further increased at the 24 h time point. Interestingly, in the nanostructure-induced neuritogenesis a strong nuclear phosphorylation of CREB (significantly stronger than the one of the canonical condition) was already present after 1 h. After 24 h this signal was reduced compared to the 1 h time point but remained still in the range of the canonical signal.

Another interesting TF candidate in this context is JNK/c-jun which is known to be downstream of integrin signaling and susceptible to geometric cues [39] and involved in neurogenic processes, e.g. neurite/axon development [59]. In keratinocytes reduced FA/stress fiber formation on soft hydrogels coincides with augmented JNK phosphorylation [60].

Congruently to the described biological effects of this TF, indeed a higher inhibitor concentration against c-jun is needed to completely block the differentiation induced by the biomaterial (indicating a higher c-jun activity) compared to the canonical NGF-stimulated condition (Fig. 8b). An interesting fact to mention is that this result is opposite to the result obtained with LPA which displays the reversed biological effect on FAs and the cytoskeleton (Additional file 3: Figure S3C) and in fact realized its inhibitory impact on nanostructure-induced neuritogenesis already at lower concentration compared to the NGF-induced one.

The results for CREB and JNK/c-jun indicate that TFs, known to be susceptible to mechanotransductive pathways and with substantial roles in neuronal differentiation, are effectively modulated by the cellular interaction with nanostructured zirconia interaction.

### Proteomic profile of nanostructure-induced neuritogenesis and mechanotransduction

**Fig. 9:**
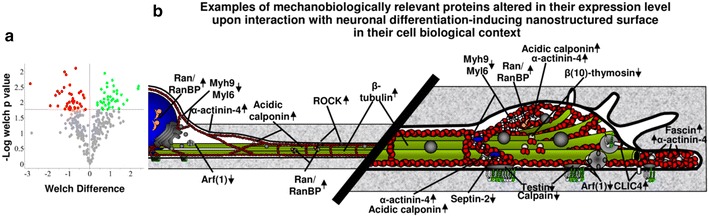
Proteomic analysis confirms the differentiation and reveals alterations of the mechanotransductive cellular status upon nanostructure/cell interaction. **a** A shotgun proteomic analysis was carried out on PC12 cells on neuritogenesis-inducing ns-Zr15 or on flat-Zr or PLL in the presence of NGF (after 24 h cell/substrate interaction). An ANOVA test was performed in order to identify the proteins that were differentially expressed. In this report, only the data comparing ns-Zr15 and flat-Zr are presented. The* colored* data points in the* volcano plot* that are located above the p value line (t test value cut off is 0.0167) correspond to the proteins that were differentially expressed in these two conditions upon treatment with large magnitude fold changes and high statistical significance. In *green* are indicated proteins that are up regulated, in* red* are the down regulated. The proteins having a fold-change less than 1.5 are shown in gray. A complete list of these proteins can be found in Additional file 4: Table S1, Additional file 5: Table S2 in the supplementaries. **b** The cartoon summarizes and visualizes the sites of action and functions of adhesome- and mechanobiologically-relevant proteins found to be altered in their expression level upon interaction with the neuritogenesis-inducing nanostructured surface (for further details see text). *Arrows* indicate up- or downregulation compared to the flat zirconia condition

In order to confirm the neuronal differentiation and to further characterize the mechanotransductive mechanism at the molecular level we performed a shotgun proteomic analysis comparing the proteome of PC12 cells grown on neuritogenesis-inducing ns-Zr15 substrates with the one of cells grown on flat-Zr and PLL (in the presence of NGF) (after 24 h cell/substrate interaction). Based on the analysis detailed in the “Methods”, 52 proteins were found upregulated or present only in cells grown on ns-Zr15, while 54 proteins were downregulated in cells on ns-Zr15 or were present only in cells on flat-Zr (Fig. 9a; Additional file 4: Table S1, Additonal file 5: Table S2).

Analyzing in detail the proteins differentially expressed, several of them reflect also at the protein level the morphologically observed differentiation processes induced by the nanostructure. In particular, the upregulation of β2-tubulin is in line with the observed induction of neuritogenesis as it represents one of the main structural components of the neurite/axon and its knockdown decreases neurite outgrowth and neuronal differentiation [61]. UCH-L1 is long-known to be upregulated in differentiated PC12 [62] and enhances neurogenesis in NPC by regulating their morphology and differentiation [63]. In congruency with the data on CREB phosphorylation (Fig. 8a), we have found several genes whose expression is known to be potentially modulated by CREB [58] and which are indeed differentially expressed in ns-Zr compared to flat-Zr (marked with crosses in Additional file 4: Tables S1, Additonal file 5: Table S2). Considering the fact that PC12 are also a neurosecretion model [64], the upregulation of the aldehyde dehydrogenase ALDH4A1 and the aspartate aminotransferase Got1, which play a role in the metabolism of the neurotransmitter glutamate (Reactome: DOI: 10.3180/REACT_13.4), is an indication of potential beginning neurosecretory activity. Moreover, also in the section below highlighting mechanotransductively relevant proteins (Fig. 9b), further proteins with prominent well-documented functions in neuronal differentiation are broadly present.

Regarding adhesion and integrin signaling, there is an evident abundance of integrin adhesome-related proteins among the up- or downregulated proteins in the PC12 cells interacting with the ns-Zr15. 7 out of 63 proteins concordantly found in three proteomic adhesome analyses [65] are highly significantly altered in their expression level. Furthermore, eight proteins are in the integrin adhesome list defined by Winograd-Katz et al. [66] (these adhesome proteins are marked in the Additional file 4: Tables S1 and Additonal file 5: Table S2 in dark grey, further proteins with roles in mechanobiological processes mentioned below are marked in light grey).

Among the adhesome proteins the downregulated protein testin is of eminent significance, taking into account the results regarding FA dynamics and actin filament bundling on the different surfaces reported in this study. Testin is a FA protein known to interact with several cytoskeletal and FA proteins, such as actin, MENA, talin, VASP and zyxin, and plays an important role in the regulation of cell spreading and migration [67]. Its downregulation causes the loss of stress fibers and a decrease in RhoA activity [68]. Furthermore, in a proteomic analysis testin was found to be one of the LIM domain proteins whose recruitment to the adhesome is myosin II-dependent and essential in the process of FA maturation. In general, LIM domain-containing proteins are emerging as key players in actin cytoskeletal- and FA-dependent cellular mechanotransductive responses [3]. In this context also the downregulation of the Class IIA myosin component MYH9 and the myosin light chain regulatory protein MYL6 are quite interesting. A decrease in their expression level is accompanied by long process formation, e.g. in fibroblasts [69].

Also the downregulated GTPase Arf1 can be found associated with FA proteins and the membrane and is thus known to be involved in FA maturation and cytoskeletal organization. In fact, it regulates the recruitment of paxillin and β1 integrin-binding partners (e.g. talin, vinculin, FAK) to the FA [70]. In addition, in the neuronal context it is also involved in the modulation of actin polymerization for synaptic plasticity [71].

Another interesting example is the upregulated α-actinin-4, a member of an actin-binding protein family which serve as protein interaction platforms and as such have versatile, in particular cytoskeleton-related functions [72], e.g. in the initial maturation phase of nascent adhesions [73]. Interestingly, in developing neurons it is involved in the distribution of f-actin [74]. Its activity is regulated by MAPK/calpain-dependent processes [72].

Calpain again can be found among the downregulated adhesome proteins. Indeed, this protease has various substrates among adhesome proteins and many tasks [75]. In the neuronal context it has a vital role in a number of axon/neurite growth cone-related processes, e.g. growth cone collapse and neurite consolidation. Its inhibition enhances neurite budding and stimulates cortactin-dependent actin polymerization [76].

In addition, there are further up- or downregulated proteins which may not be direct integrin adhesome members but nevertheless quite intriguing from the cytoskeletal and mechanotransductive perspective (and often function in close relation to integrin adhesome proteins).

The upregulated acidic calponin (calponin-3), for example, is a protein that is found to be upregulated in postmitotic neurons, localised mainly in the growth cones [77]. Acidic calponin inhibits actomyosin activity [78] and indeed its overexpression causes a re-adjustment of the organization and force balance between microtubules and actin filaments, which leads to long process formation, even in non-neuronal cells (like HEK293) [79], or elongated dendrites in hippocampal neurons [80].

Another interesting result is the upregulation of CLIC4 and fascin. CLIC4 orchestrates Rab35-dependent trafficking of β1 integrin and thereby cell adhesion [81]. Rab35 has been shown to regulate neurite outgrowth in PC12 cells, by Cdc42-dependent modulation of the actin cytoskeletal organization and cell shape [82]. It was also demonstrated that Rab35 triggers actin bundling by recruiting fascin to the plasma membrane [83]. Fascin itself is known to be a decisive factor in FA and stress fiber dynamics by inhibiting myosin II activity and slowing down FA turnover [84]. Furthermore it is an essential key protein for filopodia formation, due to its localized tight actin bundling capacity, which drives growth cone advancement [85]. In our context, CLIC4 could balance the Rab35/fascin-dependent actin bundling activity necessary for accurate neurite formation.

Four further proteins with prominent demonstrated functions in cytoskeletal organization are altered in their expression level. The upregulation of ROCK might be confusing at a first glance because the inhibitory function of ROCK/RhoA activity for the necessary reorganization of the actin cytoskeleton and membrane exocytosis for neurite budding and growth cone advancement is well-established [86, 87]. The role of ROCK/RhoA in neuritogenesis is more complex, though, its moderate and local activation is necessary to stabilize actin filaments and growth cone point contacts [46]. Furthermore it suppresses lamellipodial protrusions during axon/neurite consolidation which maintains the growth cone polarity [88]. Its actomyosin contraction-promoting activity could instead be locally controlled and diminished by the altered presence of calponin-3 and testin that both as aforementioned counteract this function. In addition, it might be reminded in this context that myosin II components are downregulated, too. Also septin-2 is downregulated, which is known to interact with myosin II serving thereby as a sort of regulatory hub to scaffold and recruit proteins that control the contractility of actomyosin [89]. In this context the downregulation of β10-thymosin is in accordance. β-thymosins bind globular monomeric actin and are therefore pivotal for the control of actin cytoskeleton dynamics and in the regulation of neuritogenesis. Indeed, their knockdown increases the outgrowth of neurites [90]. Also the upregulated Ran and RanBP1/3 are involved in cytoskeletal processes at distant sites from the nucleus. In fact, Ran knockdown results in abnormal neurite morphology because of augmented branching. Furthermore these proteins are crucial for axonal retrograde signaling [91].

Altogether, this proteomic profile of proteins altered in their expression level reflects, broadly and in a congruent manner, the differentiation events and cell biological effects described throughout the precedent paragraphs. In particular, the abundance of proteins that are involved in adhesome, cytoskeletal organization and/or cellular biomechanics is striking (Fig. 9b) and their up- or downregulation in line with their demonstrated functions in neuronal differentiation processes. Furthermore, the results have revealed some interesting otherwise maybe unrecognized or underestimated candidates for more detailed future analysis of mechanotransductive processes induced by nanoscale topography of substrates.

## Conclusions

We have characterized, in the context of neuronal differentiation, the sequence of the mechanotransductive events starting from the cell interaction with nanoscale topography and we have followed the triggered intracellular cytoskeletal/biomechanical dynamics and signaling cascades to the activation of transcription factors, eventually addressing the consequences on the cellular program.

The data were obtained with the cell line PC12 as a widely accepted and studied model for neuronal differentiation processes. The use of the PC12 cell line allowed us a broad experimental freedom whereby we were able to build a rationale picture of nanoscale topography-induced mechanotransductive processes leading to cell differentiation, and to connect them causally. This provided an in-depth understanding of how nanoscale topography induces complex mechanotransductive, molecular mechanisms that eventually modulate cell biological functions.

**Fig. 10:**
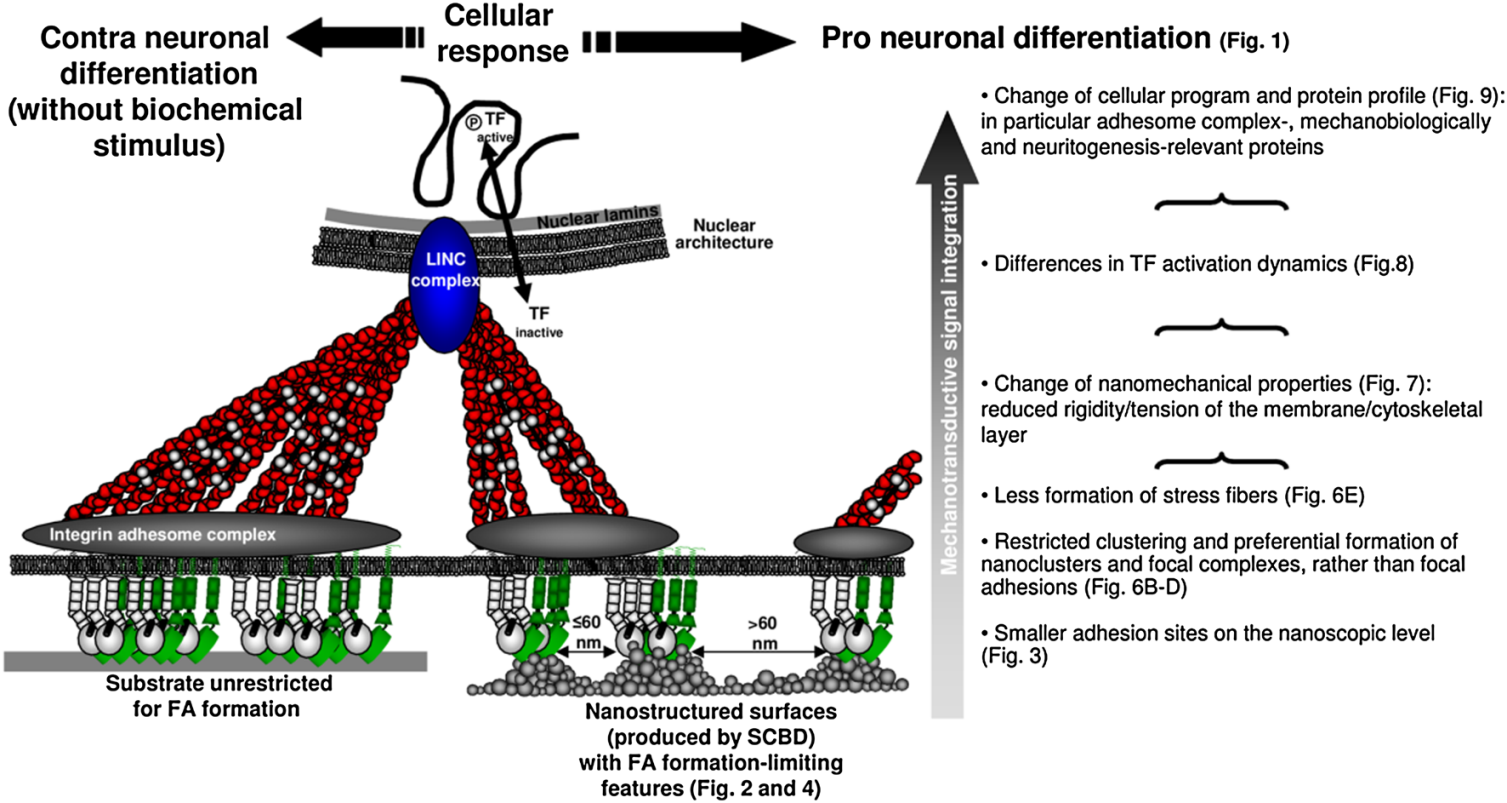
Summary and model of the molecular mechanism of SCBD-induced cell biological responses

Taken together, our results show that an adequate nanoscale surface structure, produced by SCBD of zirconia nanoparticles, has the potential to limit integrin clustering and the grade of FA formation. This alteration of the FA architecture and dynamics, enforced by the nanoscale information provided by an appropriate surface topography, feedbacks on the correlated adhesome architecture/composition and the biomechanical properties of the cell. These mechanotransductive pathways modify the activation dynamics of TFs susceptible to mechanosensitive inputs. Furthermore, the change of the cellular protein profile sets an overall cellular status eventually promoting, in this case, neuronal differentiation equivalent to the canonical NGF-induced one (summarized in Fig. 10). The indicated mechanotransductive signal integration initiated by the interaction of the cells with the neuritogenesis-inducing nanostructured surfaces was linked to the induced differentiation.

It can be speculated that the interaction of PC12 cells with a suitable nanostructured surface resembles more closely the in vivo physiological ECM configuration where neuronal cells naturally differentiate (compared to the flat PLL-coated glass or zirconia). Therefore, by the demonstrated mechanotransductive events, a biomechanical status might be set that lowers the threshold for the induction of neuronal differentiation and favors neurite budding and neurite growth cone advancement on the ns-Zr15, in this model even in the absence of a biochemical stimulus. On the flat, from a nanotopographical point of view more unnatural anti-differentiation glass-PLL surface instead the right biomechanical status has to be implemented by a cumbersome reorganization of the cytoskeleton induced by a sufficient biochemical input. The still large vinculin clusters in the PLL +NGF condition after 4 h (compared to the ns-Zr15 condition, see Fig. 6b, c) are an indication for this hypothesis. On flat-Zr not even this biochemical stimulus is sufficient, possibly due to the enhanced FA formation and cellular rigidity. Future experimentations will further address these aspects.

We are aware of the fact that results obtained with cell lines should be handled with care and that details might differ for other (neuronal/primary) cells, depending e.g. on their cell adhesion receptor profile/density and intrinsic intracellular cytoskeletal/signaling dynamics [3, 41]. These factors might also have influence on the appropriate roughness to obtain the desired biological responses. It is very likely, though, that the basics of the nanostructure-induced effects presented here, are comparable in equivalent cells. In fact, the neuronal differentiation-promoting capacity of these nanostructured zirconia surfaces is not restricted to the PC12 cell line, but is broadly confirmed by preliminary studies with the clinically more relevant cell model dissociated primary hippocampal neurons (unpublished data). Also a recent publication by Sun et al. has shown that soft biomaterials support YAP-mediated neuronal differentiation of human pluripotent stem cells into motor neurons by mechanotransductive ROCK signalling-dependent processes impacting on actomyosin contractility [92].

Altogether, this work lays a substantial cell biological foundation for the intelligent design of substrates for cell culturing based on nanostructured surfaces produced by cluster assembling that mimic more closely physiological 3D extracellular microenvironmental features. Our data suggest that the nanoscale information provided by these surfaces could have a strong potential in favoring neurogenic processes by mechanotransductive processes also in adequate primary or stem cell systems [2, 4, 9, 10]. Biophysical cues that can improve neuroinduction protocols would indeed have a significant relevance for neuroscience research (e.g. for the development of in vitro disease models [93] or neural interfaces and circuits [94]) and cell replacement strategies in neurodegenerative diseases [93].

We demonstrated that SCBD is a robust bottom-up technology for the reproducible and high-throughput fabrication of zirconia substrates with controlled nanoscale topography constituting a very effective tool to study mechanotransductive signaling. It is important to underline that the experiments reported here required a huge number of substrates (~150 Ø13 mm glass cover slips, dozens of Ø24 mm glass cover slips, several Ø40 mm glass-bottomed dishes and 76 × 26 mm microscope slides) with reproducible nanoscale roughness over a large macroscopic area. This is a serious obstacle for most of the top-down nanofabrication technologies usually employed for surface nanostructuring, in terms of fabrication time, costs and reproducibility [4, 12].

## Methods

### Substrate fabrication

Nanostructured zirconia films with controlled and reproducible nanoscale morphology were produced by supersonic cluster beam deposition (SCBD) using a deposition apparatus equipped with a pulsed microplasma cluster source (PMCS) [22].

In the PMCS an argon plasma jet ignited by a pulsed electric discharge ablates a zirconium rod. Zr atoms and ions sputtered from the target thermalize with the argon and traces of oxygen present in the condensation chamber and aggregate to form ZrO_x_ clusters. The mixture of clusters and inert gas then expands into a vacuum, through a nozzle, to form a seeded supersonic beam. The clusters carried by the seeded supersonic beam are collected on a substrate intersecting the beam trajectory (deposition rate of about 0.5–2.5 nm/min) and placed in a second vacuum chamber, thus forming a cluster-assembled film. Further oxidation of ZrO_x_ clusters takes place upon exposure to ambient atmosphere thus forming a ZrO_2_ film.

Two different batches of cluster-assembled ZrO_2_ films (called ns-Zr, hereafter) with roughness R_q_ of 15 nm (ns-Zr15) and 25 nm (ns-Zr25) were produced on round glass coverslips (Ø13 mm), microscope glass slides (76 × 26 mm area), glass-bottomed cell culture dishes (Ø40 mm) or Aclar^®^ films. As a reference we also produced flat ZrO_2_ films (R_q_ = 0.4 nm) by electron beam evaporation of a solid Zr target (flat-Zr). For the experiments, the samples with zirconia surfaces were sterilized with UV light for 10 min directly before seeding the cells on them.

Glass coverslips coated with poly-l-lysine (PLL) (Sigma-Aldrich, St. Louis, USA, Missouri) with a roughness R_q_ < 1 nm were used as standard reference substrates. For this condition, the PLL was incubated for 30 min at RT on clean glass coverslips. The coated glass was then washed twice with PBS and sterilized with UV light for 10 min. The coating procedure was performed directly before plating the cells.

### Atomic Force Microscopy characterization of substrates surface morphology

The surface morphology of cluster-assembled zirconia films and other substrates was characterized by atomic force microscopy (AFM) in air using a Multimode AFM equipped with a Nanoscope IV controller (Bruker, Billerica, USA, Massachusetts), operated in Tapping Mode. Rigid silicon cantilevers (k ≈ 40 N/m, f_0_ ≈ 300 kHz) mounting single crystal silicon tips with nominal radius 5–10 nm have been used. On each samples several 2 µm × 1 µm images were acquired with scan rate of 1 Hz and sampling resolution of 2048 × 512 points. The images were flattened by line-by-line subtraction of first and second order polynomials in order to remove artefacts due to sample tilt and scanner bow. From flattened AFM images root-mean-square surface roughness R_q_ was calculated as the standard deviation of surface heights.

In order to recognize the main asperities of the surface where cell adhesion contact points are likely to develop, we have applied to AFM topographical maps suitable thresholds on heights in order to segment the image and identify the more relevant morphological protruding features; to this purpose, height thresholds were determined for each image as the z values which maximized the number of isolated topographic features (the asperities) surviving above-threshold (details on this procedure are provided in the Additional file 2: Figure S2A–C). This choice is aimed at identifying the maximum number of asperities that a surface can offer as potential contact sites for cells. The geometrical properties of single asperities (diameter, height, radius of curvature and contact area) were determined by custom image-processing routines written in Matlab (Mathworks, Natick, USA, Massachusetts), based on the Image Processing Toolbox (details in the caption of Additional file 2: Figure S2). In particular, we assumed that each asperity can be approximately described as a spherical cap, with a given diameter at the base, height and curved area (the contact area). Eventually, we identified the potential of the asperity features to enable intracellular clustering of the adhesion spots to superior adhesion structures (such as nanoclusters, focal complexes and adhesions), applying the criterion that the separation between asperities should not exceed 60 nm, and grouping the selected asperities into clusters. This criterion is based on the ligand spacing value found to be critical for focal adhesion formation [30].

### Cell culture

PC12 (PC-12 Adh ATCC Catalog no.CRL-1721.1TM) were cultured in RPMI-1640 Medium (Sigma-Aldrich) supplemented with 10 % horse serum (HS; Sigma-Aldrich), 5 % fetal bovine serum (FBS; Sigma-Aldrich), 2 mM l-glutamine, 100 units/ml penicillin, 100 μg/ml streptomycin, 1 mM pyruvic acid (sodium salt) and 10 mM HEPES. The culture condition in the incubator (Galaxy S, RS Biotech, Irvine, UK) were maintained at 5 % CO_2_, 98 % air-humidified. For subculturing (routinely performed every 2nd–3rd day) the cells were detached from culture dishes using a 1 mM EDTA solution in HBSS (Sigma-Aldrich) or a trypsin solution (Sigma-Aldrich), centrifuged at 1000×*g* for 5 min, and re-suspended in culture medium.

### Preparation of the PC12 cells for the diverse cell biological experiments and analysis of differentiation

PC12 cells were detached with 1 mM EDTA in HBSS, centrifuged at 1000×*g* for 5 min and washed with low serum differentiation medium (RPMI plus supplements but with 1 % HS only and no FBS). Then the cells were counted with an improved Neubauer chamber and plated at a concentration of 7500 cells/ml (~4000 cells/cm^2^ in a well of a 24 well plate). If a NGF stimulus (human NGF-β from Sigma-Aldrich) was scheduled, it was added to the medium at a concentration of 50 ng/ml. In the case of a treatment with inhibitors, antibodies or other reagents, the cells were always pre-incubated in suspension for 15 min with the reagent in the indicated concentration (see figure legends) before plating. The treatment was then continued, either for the whole period of the experiment or for 1 h in the initial phase of cell/substrate interaction (as indicated in the figure legends of the single experiments). For the gradual hypoosmotic compensation experiment the cells (after 15 min preincubation in suspension) were plated on ns-Zr15 in the presence of RPMI diluted 9/1; 8,25/1,75; 7,5/2,5 and 6/4 with deionized water (always supplemented with 1 % HS). The hyperosmotic gradient ranged from 25–150 mM sucrose in 1 % HS differentiation medium. To document and quantify the morphological changes, phase contrast images were recorded with an inverted Axiovert 40 CFL microscope (Zeiss, Oberkochen, Germany) equipped with a LD A-Plan 20x/0.3 Ph1 objective (Zeiss), and then analyzed with ImageJ (NIH, New York, USA, New York).

For the differentiation rate only cells with neurites >10 μm were considered as differentiated. For the neurite length measurement, only neurites >10 μm were quantified, in case of more than one neurite, only the two longest were considered for the quantification and if the neurite branched only the longest branch was measured. Each morphological analysis comprised >500 cells for the differentiation rates and >150 neurites for the neurite outgrowth, from 2–5 independent experiments.

### Antibodies, reagents and inhibitors for cell biological experiments

The reagents used in work were the following. Antibodies or fluorescence reagents: 4B4 (Beckman Coulter) and K20 (Santa Cruz Biotechnology, Santa Cruz, USA, California) against β1 integrin, 87G3 antibody against p-CREB (Cell signaling, Danvers, USA, Massachusetts), hVin-1 antibody against vinculin (Sigma-Aldrich), HOECHST 33342 (Molecular Probes (Thermo Fischer Scientific), Waltham, USA, Massachusetts), TRITC-Phalloidin (Sigma-Aldrich). Inhibitors: blebbistatin, cytochalasin D, EHop-016, GW441756, lysophosphatidic acid, methyl-β-cyclodextrin, nocodazole, PF-573228, SP600125, U1026, Y27632 (all Sigma-Aldrich).

### Transmission electron microscopy analysis of nanoscopic adhesion regions

Flat and cluster-assembled zirconia films have been produced on Aclar^®^ films. 24 h post seeding, cells were fixed in 0.1 M Na cacodylate buffer with 1.2 % glutaraldehyde for 1 h at RT and heavy metal stained as described in Deerinck et al. [95], with minor modifications. Briefly, cells were incubated for 1 h on ice in a solution containing 1.5 % potassium ferrocyanide, 2 % osmium tertroxide and 2 mM CaCl_2_ in 0.1 M Na cacodylate buffer, rinsed with ddH_2_O and incubated with a thiocarbohydrazide solution (10 mg/ml in ddH_2_O) for 20 min at RT. Cells were rinsed again in ddH_2_O and exposed to 2 % osmium tetroxide in ddH2O for 30 min, rinsed and stained by a saturated solution of uranyl acetate in ddH_2_O for 45 min. Cells were then dehydrated by an EtOH series and embedded in Epon resin.

Ultrathin sections were cut using an UltraCut6 ultramicrotome (Leica Microsystems GmbH, Wetzlar, Germany), collected on Formvar coated copper slot grids and imaged with a Tecnai G2 transmission electron microscope (FEI, Hillsboro, USA, Oregon). The images were analyzed with ImageJ (NIH). In order to define the dimension of nanoscopic adhesion sites, profiles of the cell membranes and the substrates were manually traced and both converted into collection of XY coordinates. Using the coordinates, the distance between of every membrane point and the substrate was defined as the shortest segment connecting that point to substrate profile out of all the possible ones. Taking into account the structure and extracellular length of extended integrins and the position of their ligand interaction site [29], we defined the adhesion sites of the cell with the substrate as sections of the membrane where the distance with the substrate is equal or below 15 nm. Respecting this criterion, we eventually measured the length of the adhesion contact regions. In total, for global statistics 164 adhesion regions on cluster-assembled zirconia and 120 on flat zirconia were analyzed surfaces, obtained from images deriving from two independent experiments.

### Immunofluorescence imaging

Cells were fixed with 4 % PFA/PBS, permeabilized with 0.2 % Triton X-100/PBS, blocked with 3 % BSA/PBS and incubated with the primary antibody for at least 1 h at RT (or alternatively overnight at 4 °C) and in humid conditions, the secondary antibody was incubated at RT for maximum 1 h. The actin cytoskeleton was stained with TRITC-phalloidin (Sigma-Aldrich) which was added to the secondary antibody. Optionally HOECHST (Molecular Probes) staining was performed to mark the nucleus. If the samples were mounted, this was done with ProLong^®^ Gold antifade (Molecular probes).

### Analysis of focal adhesions and actin filaments by Total Internal Reflection Fluorescence (TIRF) microscopy

PC12 cells were seeded on the different substrates (on Ø24 mm glass cover slips). At the indicated time points (30 min, 1, 4 and 24 h) the cells were fixed and labeled for vinculin and f-actin following the immunofluorescence imaging protocol described above. The images were recorded with a Leica AM TIRF MC system using a Leica HCX PL APO 63X NA 1.47 objective (Leica). To visualize the vinculin clusters, integrated 488 nm laser lines and Andor iXon DU-885 camera (Andor Technology, Belfast, UK) was used. The image recording was done with a laser incident angle of 74° allowing a penetration depth of almost 250 nm. The images were elaborated with ImageJ following a recently described method [96] to analyze the area and number of clusters per cell. In total, for global statistics 722-3678 clusters from 16–34 cells were analyzed from three independent experiments. The f-actin instead was imaged in epifluorescence mode. Here the cytoskeletal organization of the cells was categorized in three categories: (1) no detectable presence of actin bundles/stress fibers, (2) 1–10 distinct actin bundles/stress fibers and (3) >10 distinct actin bundles/stress fibers.

### Analysis of the mechanical properties of living PC12 cells by AFM

Cells were plated in the standard experimental conditions on Ø40 mm glass-bottom cell culture dishes (Willco Wells, Amsterdam, Netherlands), covered either with flat or cluster-assembled zirconia films or coated with PLL. During the AFM measurements the temperature of the medium was maintained at 37 °C by a custom built thermostatic fluid cell (for details see Fig. 12 and Appendix C1 of Ref. [97]). 25 mM HEPES buffer was added to keep the physiological pH of the medium.

Combined topographical and mechanical AFM imaging was performed with a Bioscope Catalyst AFM (Bruker) operated in *force volume* mode by collecting series of force vs distance curves, according to established protocols [97, 98]. We used a monolithic borosilicate glass probe consisting in a micrometer-sized spherical glass bead with radii R in the range of 4500–5500 nm attached to silicon cantilevers with elastic constant k = 0.2–0.3 N/m [99]. Each force volume consists in an array of 64 × 64 force vs distance curves recorded across the region occupied by a single cell. All measurements were performed with the following parameters: ramp length *L* = 5 μm; approaching speed *v*_*appr*_ = 43.4 μm/s; ramp frequency *f* = 7.1 Hz; 2048 points per curve. The lateral scan size varied between 50 × 50 μm and 100 × 100 μm, depending on the dimension of the cell.

Data processing of force volumes was carried out in Matlab (Mathworks) environment using custom-built routines [97]. The local height of the sample and the local effective Young’s modulus can be extracted by single force curves; by these means topographic and elastic maps of the sample can be acquired in one-to-one correspondence. The values of the Young’s moduli were extracted by fitting the Hertz model to experimental data [97]. A finite-thickness correction was applied and the force curves linearized in order to identify the presence of multiple elastic regimes inside the cell, and more generally the upper limit of validity of the Hertz model. Following this procedure, the effective Young’s modulus of the cell was typically evaluated by fitting the 0–40 % range of the total local indentation. The cumulative distributions of Young’s moduli of the cells turned out to be the envelope of a few (typically two–three) lognormal modes, originating from micro-scale domains that the AFM probe was able to resolve. Multi-Gaussian fit in semilog10 scale allowed identifying the peak value E’ and the geometric standard deviation ${{\upsigma }}_{\text{g}}^{10}$ of each lognormal mode; from these values the median value E_med_ and the standard deviation of the median σ_med_ were calculated for all modes as ${\text{E}}_{\text{med}} = 10^{{{\text{E}}^{'} }}$ and ${{\upsigma }}_{\text{med}} = \sqrt {{{\uppi }}/2} {\text{E}}_{\text{med}} {{\upsigma }}_{\text{g}}^{10} /\sqrt {\text{N}}$ [100], N being the number of force curves in each mode. The effective rigidity of cells was obtained as the weighted average of median values: ${\text{E}} = \mathop \sum \limits_{\text{i}} {\text{f}}_{\text{i}} {\text{E}}_{{{\text{med}},{\text{i}} }}$, using the fraction f_i_ = N_i_/N_tot_ of force curves in each mode as weight; the total error σ_E_ associated to E was calculated by summing in quadrature the propagated error of the medians ${{\upsigma }} = \sqrt {\mathop \sum \limits_{\text{i}} {\text{f}}_{\text{i}}^{2} {{\upsigma }}_{{{\text{med}},{\text{i}}}}^{2} }$ and an effective instrumental relative error σ_instr_ = 10 %: ${{\upsigma }}_{\text{E}} = \sqrt {{{\upsigma }}_{\text{instrum}}^{2} {\text{E}}^{2} + {{\upsigma }}^{2} }$. Finally, the average median values of the Young’s Modulus of all cells belonging to the same condition have been evaluated; the corresponding error has been calculated as the standard deviation of the mean summed in quadrature with the propagated σ_E_. The statistical significance of differences between Young’s moduli of cells from different culture conditions has been evaluated applying the two-tails t Test. 6–8 cells have been measured for each condition derived from two to three independent experiments.

### Analysis of CREB phosphorylation by confocal microscopy

PC12 cells were plated on the indicated substrates and fixed with 4 % PFA at the different time points. The immunofluorescence staining was done as described above. For this experiment it was stained for Ser 133-phosphorylated CREB (antibody 87G3 from Cell signaling), f-actin (Phalloidin) and the nucleus (HOECHST). To quantify the specific nuclear Ser133 phospho-CREB signal, the average-projection signal of the nuclear Ser133 p-CREB (the nuclear region was determined from the max-projection of the HOECHST staining) was set in relation to the average-projection signal of the total cell (total cell region was determined from the outlines of max-projection of f-actin staining). The confocal images were recorded with a Leica confocal microscopy TCS SP2 (Leica). In total, for global statistics the signals of 33–76 cells were analyzed from two independent experiments.

### Proteomic analysis

After 24 h on the indicated substrates the cells were scratched from the substrates with a cell scraper (TPP, Trasadingen, Switzerland) (on ice) in the presence of icecold PBS supplemented with protease inhibitor (Roche, Basel, Switzerland).

After reduction and derivatization, the proteins were digested with trypsin sequence grade trypsin (Roche) for 16 h at 37 °C using a protein:trypsin ratio of 1:20. LC-ESI-MS/MS analysis was performed on a Dionex UltiMate 3000 HPLC System with a PicoFrit ProteoPrep C18 column (200 mm, internal diameter of 75 μm) (New Objective, Woburn, USA, Massachusetts). Gradient: 1 % ACN in 0.1 % formic acid for 10 min, 1–4 % ACN in 0.1 % formic acid for 6 min, 4–30 % ACN in 0.1 % formic acid for 147 min and 30–50 % ACN in 0.1 % formic for 3 min at a flow rate of 0.3 μl/min. The eluate was electrosprayed into an LTQ Orbitrap Velos (Thermo Fisher Scientific) through a Proxeon nanoelectrospray ion source (Thermo Fisher Scientific). The LTQ-Orbitrap was operated in positive mode in data-dependent acquisition mode to automatically alternate between a full scan (m/z 350–2000) in the Orbitrap (at resolution 60,000, AGC target 1,000,000) and subsequent CID MS/MS in the linear ion trap of the 20 most intense peaks from full scan (normalized collision energy of 35 %, 10 ms activation). Isolation window: 3 Da, unassigned charge states: rejected, charge state 1: rejected, charge states 2+, 3+, 4+: not rejected; dynamic exclusion enabled (60 s, exclusion list size: 200). Four technical replicate analyses of each sample were performed. Data acquisition was controlled by Xcalibur 2.0 and Tune 2.4 software (Thermo Fisher Scientific).

Mass spectra were analyzed using MaxQuant software (version 1.3.0.5). The initial maximum allowed mass deviation was set to 6 ppm for monoisotopic precursor ions and 0.5 Da for MS/MS peaks. Enzyme specificity was set to trypsin, defined as C-terminal to arginine and lysine excluding proline, and a maximum of two missed cleavages were allowed. Carbamidomethylcysteine was set as a fixed modification, N-terminal acetylation and methionine oxidation as variable modifications. The spectra were searched by the Andromeda search engine against the rat Uniprot sequence database (release 29.05.2013). Protein identification required at least one unique or razor peptide per protein group. Quantification in MaxQuant was performed using the built-in XIC-based label free quantification (LFQ) algorithm using fast LFQ [101]. The required false positive rate was set to 1 % at the peptide and 1 % at the protein level against a concatenated target decoy database, and the minimum required peptide length was set to six amino acids. Statistical analyses were performed using the Perseus software (version 1.4.0.6, www.biochem.mpg.de/mann/tools/). Only proteins present and quantified in at least 3 out of 4 technical repeats were considered as positively identified; 748, 720 and 764 proteins were identified in ns-Zr15, flat-Zr and PLL +NGF, respectively; 18 proteins were exclusively expressed in ns-Zr15, 14 proteins in flat-Zr, and 26 proteins in PLL +NGF. An ANOVA test (false discovery rate 0.05) was carried out to identify proteins differentially expressed among the three conditions: 286 out of 666 common proteins differ with statistical significance and were selected for further analyses. In particular, for the purpose of the present report, we focused only on the differential proteomics between cells on ns-Zr15 in comparison to cells on flat-Zr in order to better understand the effect of the surface nanotopography. Differential expression was considered as significant if (1) a protein was present only in ns-Zr15 or flat-Zr or (2) its normalized (according to the LFQ algorithm) intensity resulted statistical different as calculated by Post Hoc Bonferroni test (t test cut-off at p value = 0.0167) (Fig. 9a).

## Additional files

10.1186/s12951-016-0171-3 In this panel representative phase contrast images of cells after the different, indicated treatments are shown (examples of the condition without any treatment are shown in Fig. 1a). Furthermore a close-up from differentiated cells in the ns-Zr15 (-NGF) condition, taken from Fig. 1a, is shown to illustrate the typical features of neuritogenesis.
10.1186/s12951-016-0171-3 Procedure for the identification of asperity pattern from AFM images and data analysis. Height thresholds (**B**) are set for each topographic map (**A** for a representative one) and the points in the image with heights above threshold are retained. Each threshold value therefore determines a pattern of isolated nano-islands (asperities) consisting in connected sets of pixels (**C**). The best asperity pattern is determined by the threshold value maximizing the number of asperities. This choice is based on the objective to identify the maximum number of asperities that a surface can offer as potential contact sites for cells. For this purpose we adopted a statistical approach based on the observation that, as the height threshold is lowered from the topmost level, the number of above-threshold asperities tends to increase, then at a critical threshold it stabilizes, then it decreases again since asperities start merging at their bases. The optimal threshold is therefore the one that selects the more numerous and larger asperities. The asperities can be identified and labeled one by one, and their morphological parameters (diameter, height, radius of curvature, contact area, volume) are calculated. In particular, assuming that asperities are described with good accuracy by the spherical cap geometry [99], the contact area A of the asperity is calculated as A = π[(D/2)^2^ + h^2^], where D and h are the asperity diameter and height, respectively. The mean separation d between asperities is estimated based on the calculated average area per asperity, assuming each asperity occupies a square of area d^2^, so that the centers of two adjacent asperities are separated by d (we have detected about 2000–3000 asperities on both ns-ZrO_2_ samples).
10.1186/s12951-016-0171-3 Inhibition of principal mediators and cellular structures/processes involved in integrin signaling. Considering the importance of integrin signaling in both, the canonical and the nanostructure-induced neuritogenesis, we further examined prominent mediators and cellular structures/processes involved in the integrin pathway, cytoskeletal organization and neuritogenesis. The experimental set-up is the same as in Fig. 5, with the difference that here, the cells were incubated with the inhibitor only for the 1st h after plating. This treatment turned out to be sufficient for efficient inhibition but minimized collateral, cytotoxic effects of the inhibitors. The impact of these inhibitors on canonical and nanostructure-induced neuritogenesis is summarized in the graphs (representing the global statistics of two or three independent experiments, n: >500 cells, >150 neurites). **A** The graph displays the impact of inhibitors against prominent mediators of the integrin signaling; FAK (PF-573228 50 μM), Rac1 (EHop-016 2.5 μM), and ROCK (Y27632 10 μM). **B** In this graph the results for treatments with cytochalasin D (10 µM), nocodazole (1 mM), blebbistatin (50 µM) and methyl-β-cyclodextrin (5 mM) are shown. **C** The graph summarizes the impact of different concentrations (5 and 10 µM) of lysophosphatic acid (LPA) on canonical or nanostructure-induced neuritogenesis. **D** The graph displays the impact of a hyperosmotic gradient on the nanostructure-induced neuritogenesis (the same experimental set-up as in Fig. 7c). Focal adhesion kinase (FAK) and Rac1 are known to be crucial in the regulation of cytoskeletal dynamics which drive neurite outgrowth [37, 46, 49]. Indeed, the impairment of FAK and Rac1 function abolished the nanostructure-induced neuritogenesis to a similar extent as the canonical one (Additional file 2: Figure S2D). Also the disintegration of lipid rafts (cholesterol-rich membrane compartments involved in signaling events and cytoskeletal organization, particularly also in the context of neuritogenesis [102]) by methyl-β-cyclodextrin impeded both, canonical and nanostructure-induced neurite outgrowth (Additional file 2: Figure S2B). The same was true for the inhibition of actin and tubulin polymerization by cytochalasin D, or respectively nocodazole (Additional file 2: Figure S2B). The latter one even impaired the low basal differentiation congruent with the importance of tubulin polymerization for neurite budding [103]. Blocking of ROCK (inh.: Y27632) instead did not alter the eventual formation of neurites (or even slightly increased it) (Additional file 2: Figure S2A) but at the contrary clearly accelerated the initial budding in both cases (not shown). The negative regulation of neurite budding by the RhoA/ROCK pathway has been shown in many publications (e.g. [46, 86, 87]). Congruently, also reducing the affinity of myosin II to actin via blebbistatin had the same effect (**B**). Representative images of all conditions can be found in Additional file 1: Figure S1.
10.1186/s12951-016-0171-3 Proteomic data for upregulated proteins. Proteins upregulated (compared to flat-Zr) or present only in cells grown on ns-Zr15. Adhesome proteins and proteins with roles in mechanobiological processes are marked in dark and light grey, respectively.
10.1186/s12951-016-0171-3 Proteomic data for downregulated proteins. Proteins downregulated in cells on ns-Zr15 or were present only in cells on flat-Zr. Adhesome proteins and proteins with roles in mechanobiological processes are marked in dark and light grey, respectively.

## Abbreviations

AFM: atomic force microscopy
a.u.: arbitrary units
av: average
BDR: ballistic deposition regime
CREB: cAMP response element-binding protein
ECM: extracellular matrix
Erk: extracellular-signal-regulated kinase
FA: focal adhesion
f-actin: filamentous actin
FBS: fetal bovine serum
FC: focal complex
flat-Zr: flat zirconia
h: hour
HS: horse serum
JNK: c-Jun N-terminal kinases
kPa: kilo pascal
LPA: lysophosphatidic acid
MAD: median absolute deviation
MAPK: mitogen-activated protein kinase
MBC: methyl-β-cyclodextrin
mM: millimolar
na: not analyzable
NGF: nerve growth factor
NO: nitric oxide
ns-Zr: nanostructured zirconia
p-CREB: phosphorylated CREB
PLL: poly-l-lysine
PMCS: pulsed microplasma cluster source
SCBD: supersonic cluster beam deposition
SD: standard deviation
stdev: standard deviation
TEM: transmission electron microscopy
TF: transcription factor
TIRF: total internal reflection fluorescence (microscopy)
TrkA: tropomyosin receptor kinase A, also known as high affinity nerve growth factor receptor
ROCK: Rho-associated protein kinase
Rq: root-mean-square surface roughness
RTK: receptor tyrosine kinase
