# Supplementary figures and images for: Conversion of nanoscale topographical information of cluster-assembled zirconia surfaces into mechanotransductive events promotes neuronal differentiation
Source: J Nanobiotechnology. 2016 Mar 9;14:18. doi: 10.1186/s12951-016-0171-3 (PMC4784317; doi:10.1186/s12951-016-0171-3)

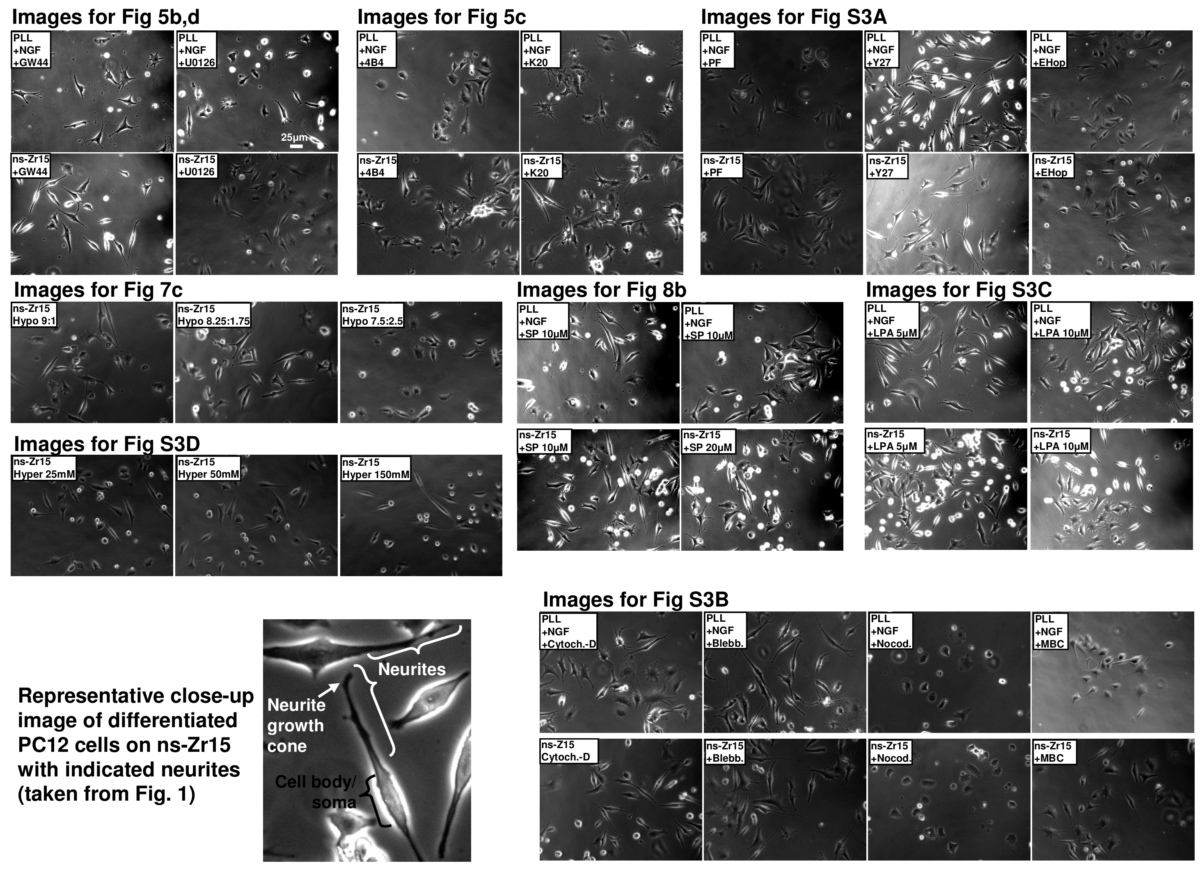

Supplement: Supplementary file 1 — 10.1186/s12951-016-0171-3 In this panel representative phase contrast images of cells after the different, indicated treatments are shown (examples of the condition without any treatment are shown in Fig. 1a). Furthermore a close-up from differentiated cells in the ns-Zr15 (-NGF) condition, taken from Fig. 1a, is shown to illustrate the typical features of neuritogenesis. [file 12951_2016_171_MOESM1_ESM.tif]

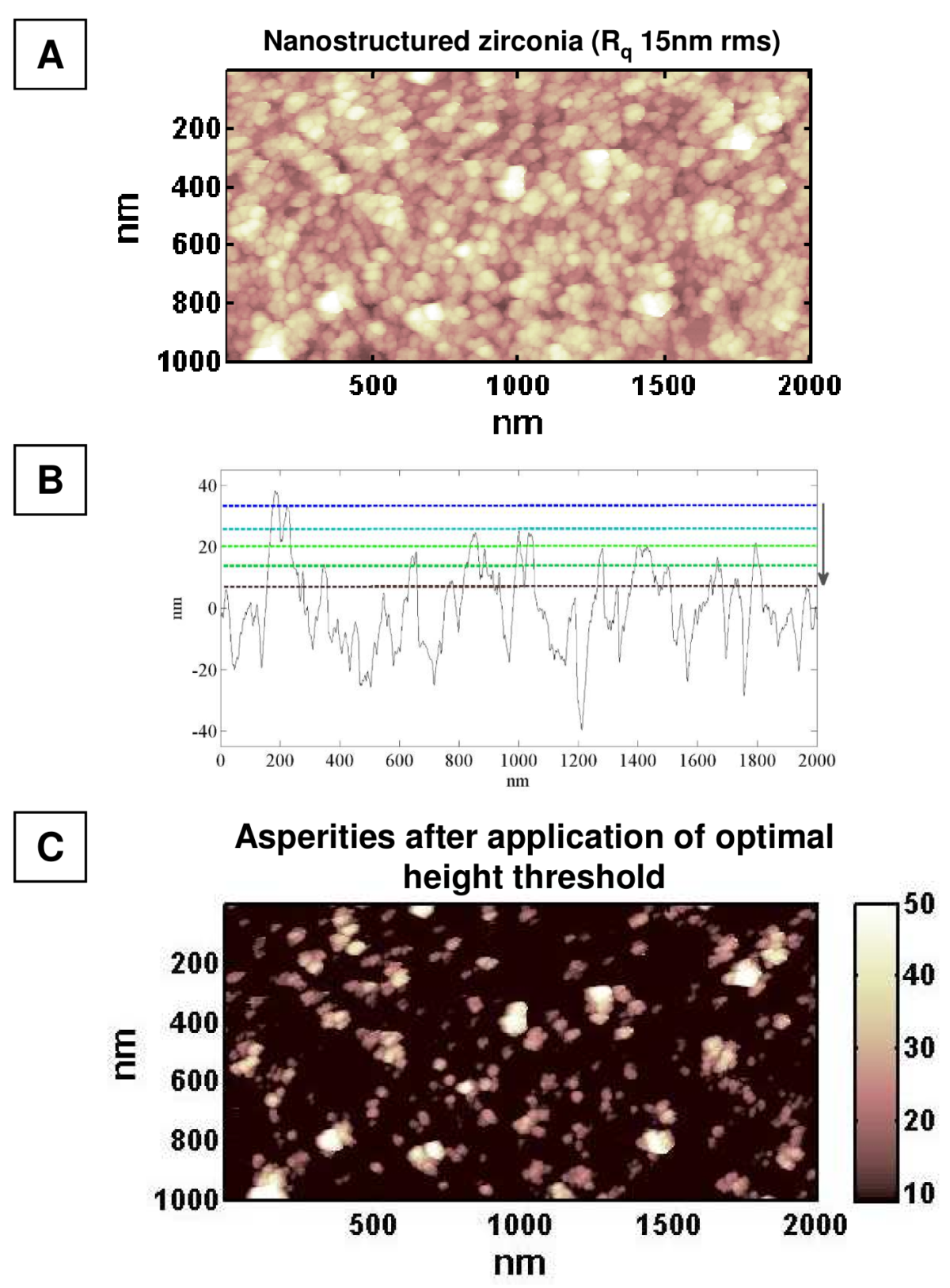

Supplement: Supplementary file 2 — 10.1186/s12951-016-0171-3 Procedure for the identification of asperity pattern from AFM images and data analysis. Height thresholds (B) are set for each topographic map (A for a representative one) and the points in the image with heights above threshold are retained. Each threshold value therefore determines a pattern of isolated nano-islands (asperities) consisting in connected sets of pixels (C). The best asperity pattern is determined by the threshold value maximizing the number of asperities. This choice is based on the objective to identify the maximum number of asperities that a surface can offer as potential contact sites for cells. For this purpose we adopted a statistical approach based on the observation that, as the height threshold is lowered from the topmost level, the number of above-threshold asperities tends to increase, then at a critical threshold it stabilizes, then it decreases again since asperities start merging at their bases. The optimal threshold is therefore the one that selects the more numerous and larger asperities. The asperities can be identified and labeled one by one, and their morphological parameters (diameter, height, radius of curvature, contact area, volume) are calculated. In particular, assuming that asperities are described with good accuracy by the spherical cap geometry [99], the contact area A of the asperity is calculated as A = π[(D/2)2 + h2], where D and h are the asperity diameter and height, respectively. The mean separation d between asperities is estimated based on the calculated average area per asperity, assuming each asperity occupies a square of area d2, so that the centers of two adjacent asperities are separated by d (we have detected about 2000–3000 asperities on both ns-ZrO2 samples). [file 12951_2016_171_MOESM2_ESM.tif]

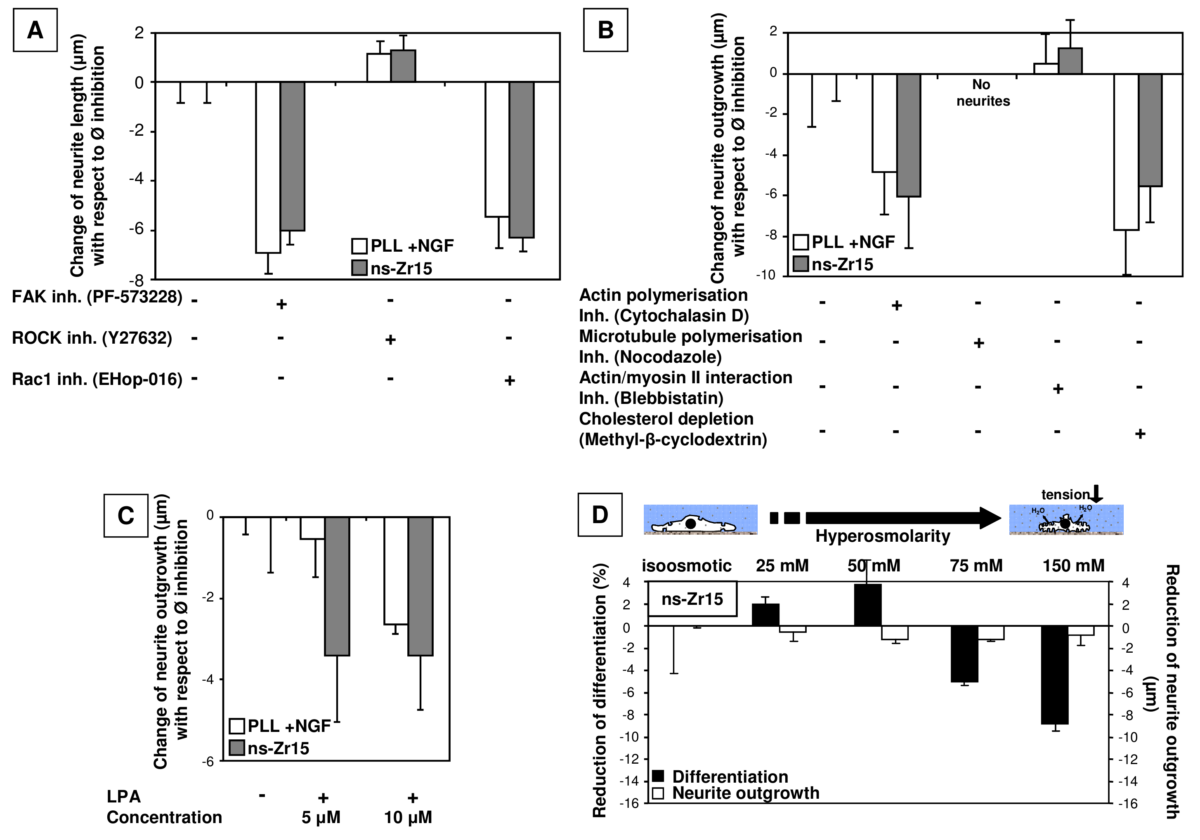

Supplement: Supplementary file 3 — 10.1186/s12951-016-0171-3 Inhibition of principal mediators and cellular structures/processes involved in integrin signaling. Considering the importance of integrin signaling in both, the canonical and the nanostructure-induced neuritogenesis, we further examined prominent mediators and cellular structures/processes involved in the integrin pathway, cytoskeletal organization and neuritogenesis. The experimental set-up is the same as in Fig. 5, with the difference that here, the cells were incubated with the inhibitor only for the 1st h after plating. This treatment turned out to be sufficient for efficient inhibition but minimized collateral, cytotoxic effects of the inhibitors. The impact of these inhibitors on canonical and nanostructure-induced neuritogenesis is summarized in the graphs (representing the global statistics of two or three independent experiments, n: >500 cells, >150 neurites). A The graph displays the impact of inhibitors against prominent mediators of the integrin signaling; FAK (PF-573228 50 μM), Rac1 (EHop-016 2.5 μM), and ROCK (Y27632 10 μM). B In this graph the results for treatments with cytochalasin D (10 µM), nocodazole (1 mM), blebbistatin (50 µM) and methyl-β-cyclodextrin (5 mM) are shown. C The graph summarizes the impact of different concentrations (5 and 10 µM) of lysophosphatic acid (LPA) on canonical or nanostructure-induced neuritogenesis. D The graph displays the impact of a hyperosmotic gradient on the nanostructure-induced neuritogenesis (the same experimental set-up as in Fig. 7c). Focal adhesion kinase (FAK) and Rac1 are known to be crucial in the regulation of cytoskeletal dynamics which drive neurite outgrowth [37, 46, 49]. Indeed, the impairment of FAK and Rac1 function abolished the nanostructure-induced neuritogenesis to a similar extent as the canonical one (Additional file 2: Figure S2D). Also the disintegration of lipid rafts (cholesterol-rich membrane compartments involved in signaling events and cytoskeletal organiza [file 12951_2016_171_MOESM3_ESM.tif]
